# SSU rDNA Divergence in Planktonic Foraminifera: Molecular Taxonomy and Biogeographic Implications

**DOI:** 10.1371/journal.pone.0104641

**Published:** 2014-08-13

**Authors:** Aurore André, Frédéric Quillévéré, Raphaël Morard, Yurika Ujiié, Gilles Escarguel, Colomban de Vargas, Thibault de Garidel-Thoron, Christophe J. Douady

**Affiliations:** 1 Centre National de la Recherche Scientifique, Unité Mixte de Recherche 5276: Laboratoire de Géologie de Lyon: Terre, Planètes, Environnement, Université Lyon 1, Villeurbanne, France; 2 Centre National de la Recherche Scientifique, Unité Mixte de Recherche 6112: Laboratoire de Planétologie et de Géodynamique - Bioindicateurs Actuels et Fossiles, Université d'Angers, Angers, France; 3 Zentrum für marine Umweltwissenschaften MARUM, Universität Bremen, Bremen, Germany; 4 Department of Biology, Shinshu University, Matsumoto, Japan; 5 Centre National de la Recherche Scientifique, Unité Mixte de Recherche 7144: Evolution des Protistes et des Ecosystèmes Pélagiques, Université Pierre et Marie Curie-Station Biologique de Roscoff, Roscoff, France; 6 Centre National de la Recherche Scientifique, Centre de Recherche et d'Enseignement de Géosciences de l'Environnement, Université Aix-Marseille, Aix-en-Provence, France; 7 Centre National de la Recherche Scientifique, Unité Mixte de Recherche 5023: Ecologie des Hydrosystèmes Fluviaux, Université Lyon 1, Villeurbanne, France; 8 Institut Universitaire de France, Paris, France; Institute of Biochemistry and Biology, Germany

## Abstract

The use of planktonic foraminifera in paleoceanography requires taxonomic consistency and precise assessment of the species biogeography. Yet, ribosomal small subunit (SSUr) DNA analyses have revealed that most of the modern morpho-species of planktonic foraminifera are composed of a complex of several distinct genetic types that may correspond to cryptic or pseudo-cryptic species. These genetic types are usually delimitated using partial sequences located at the 3′end of the SSUrDNA, but typically based on empirical delimitation. Here, we first use patristic genetic distances calculated within and among genetic types of the most common morpho-species to show that intra-type and inter-type genetic distances within morpho-species may significantly overlap, suggesting that genetic types have been sometimes inconsistently defined. We further apply two quantitative and independent methods, ABGD (Automatic Barcode Gap Detection) and GMYC (General Mixed Yule Coalescent) to a dataset of published and newly obtained partial SSU rDNA for a more objective assessment of the species status of these genetic types. Results of these complementary approaches are highly congruent and lead to a molecular taxonomy that ranks 49 genetic types of planktonic foraminifera as genuine (pseudo)cryptic species. Our results advocate for a standardized sequencing procedure allowing homogenous delimitations of (pseudo)cryptic species. On the ground of this revised taxonomic framework, we finally provide an integrative taxonomy synthesizing geographic, ecological and morphological differentiations that can occur among the genuine (pseudo)cryptic species. Due to molecular, environmental or morphological data scarcities, many aspects of our proposed integrative taxonomy are not yet fully resolved. On the other hand, our study opens up the potential for a correct interpretation of environmental sequence datasets.

## Introduction

Fossil shells of planktonic foraminifera constitute one of the most informative archive of biodiversity changes which are used as a proxy to reconstruct past ocean conditions [1]. Since these reconstructions are empirically derived from species-specific calibrations between extant environmental parameters and the abundance or chemical composition of shells of modern individual species, they require an accurate taxonomic consistency and a precise assessment of the biogeography and ecology of species [2], [3], [4]. Yet, very little is known about the biology of planktonic foraminifera [5], [6]. Consequently, following the paleontological use, the taxonomy of living species has been solely defined on the basis of diagnostic characters of their shells (morpho-species concept), mostly described from fossil type specimens extracted from sediments [7], [8].

Molecular analyses have shown that the morphological taxonomy in planktonic foraminifera underestimates biodiversity (for a review, see [9]). Small sub-unit ribosomal DNA (SSU rDNA) sequences are usually used for phylogeny involving high taxonomic ranks (e.g., [10]), but extensive single-cell sequencing revealed that foraminifera SSU rDNA sequences, compared to other eukaryotes, are highly divergent due to their rapid evolutionary rate and their long-fragment insertions [11]. In planktonic foraminifera, the SSU rDNA region appears to be a suitable marker for studying genetic diversity within and among closely related species [12], [13], [14], [15]. Thus, the large majority (∼80%) of the characterized planktonic foraminiferal sequences are localized within a 1,200 bp-long region at the 3′ end of the SSU rDNA [9], [16], [17], [18], [19]. ITS rDNA sequences have also been obtained for the three morpho-species *Truncorotalia truncatulinoides* [20], *Globoconella inflata* [21] and *Globigerinoides sacculifer* [22]. Although ITS rDNA barcodes are more commonly used than SSU rDNA to assess species-level diversity (e.g., [23], [24]), foraminiferal ITS rDNA evolves at such fast rates that even sequences from closely related morpho-species cannot be aligned [22]. This characteristic is unusual in eukaryotes (e.g., [25], [26]) and prevents the use of alignment methods involving sequences from different morpho-species. In addition, mitochondrial genes such as COI, which are the most utilized species-level barcodes in animals, have not yet been PCR-amplified and sequenced from foraminifera. As a consequence, and because species delimitations relying on genetic distances and/or phylogenetic analyses should be based on the same genetic marker to insure that they correspond to the same taxonomic level (e.g., [27], [28]), SSU rDNA is still the most widely used marker for putative species delimitation in planktonic foraminifera. Within the 25 morpho-species for which rDNA sequences are currently available, 54 genetic types, variously labeled “genotypes”, “types”, “subtypes” or “cryptic species” have been published so far (NCBI database, January 2013). Apart from one exception (*G. sacculifer*, [22]), all studied morpho-species include two to seven distinct genetic types [9], [16], [17], [19], [21], [29], several of which exhibiting a distinct biogeography and/or ecology [18], [20], [30], [31], [32], [33], [34], [35]. Molecular clocks and biogeographic patterns further suggested that these genetic types have been reproductively isolated for a significant amount of time and should be considered as cryptic or pseudo-cryptic species when subtle differences in shell morphology were additionally detected [21], [29], [36], [37], [38].

Nevertheless, a number of genetic types within a morpho-species, or a number of “subtypes” within a genetic type, exhibit partial or even total sympatric distributions and/or similar shell morphologies [9], [18], [29], [38], [39]. Many proposed genetic types were defined exclusively on the basis of genetic differences and phylogenetic criteria involving only a single or a few closely related morpho-species, without any additional DNA-independent investigation that may help validating their species status. Such difficulties, associated with the rapid increase of SSU rDNA sequences available, calls for the development of a standardized approach to rationalize species delimitation in planktonic foraminifera. Definitions of the published genetic types over the last 15 years have been optimized on the basis of various phylogenetic inferences mostly applied to isolated morpho-species, because of the high and variable SSU rDNA substitution rates, which induce highly ambiguous sequence alignments when datasets include loosely related species [17]. The resulting estimation of cryptic diversity thus relies on divergent working hypotheses. Furthermore, and because of the development of various PCR amplification strategies, sequences available are heterogeneous in length and cover different parts of the 3′ end of the SSU region. Altogether this explains why no standardized molecular procedure and threshold have ever been assessed in the case of planktonic foraminifera.

A first quantitative approach of species delimitation using a clustering method has been attempted by [40]. Although several morpho-species were not successfully separated (e.g., *Neogloboquadrina dutertrei* and *Pulleniatina obliquiloculata*), the resulting optimized taxonomic units were mostly in agreement with those determined based on a morphological taxonomy. On the other hand, no more than two genetic types were delimitated within a reduced number of morpho-species (*Hastigerina pelagica*, *Turborotalia quinqueloba*, *Gobigerinella siphonifera* and *Globigerinita uvula*) and the attempt failed to separate some highly divergent genetic types now recognized as pseudo-cryptic species (e.g., those of *Orbulina universa*; [30], [37]). In our study, based on 1352 SSU rDNA sequences either available online (NCBI query portal, January 2013) or newly obtained from living single foraminifera collected in the world oceans, we attempt to design a single and objective approach for species delimitation. We first evaluate the consistency of already defined genetic types using patristic distances calculations. Such evaluation is based on the assumption that within a given morpho-species, the genetic types validate a species status as soon as distance values are smaller within than among genetic types [41], [42]. This approach has limitations because 16 of the previously defined genetic types are known from only one SSU rDNA sequence, preventing any calculation of distances within genetic types. Furthermore, sequences that lack genetic type assignation in sequence databases such as GenBank, or for which a genetic type could not be inferred from original publication, cannot be considered for patristic distance calculations. Consequently, we test two independent and complementary automatic methods for molecular operational taxonomic unit (MOTU) delimitations: the Automatic Barcode Gap Discovery (ABGD, [42]), which allows calculation of genetic distances within and among genetic types delimitated according to each possible species-level threshold, and the General Mixed Yule Coalescent (GMYC, [43]), which uses phylogenetic trees to identify transitions from coalescent to speciation branching patterns. These methods provide alternative delimitations and offer the opportunity to analyze sequences that lack former assignation of their genetic type. Finally, these alternative delimitations are confronted in an attempt to connect SSU rDNA sequences to identified genuine species. Since the resulting molecular taxonomy we propose here may have implications for our knowledge of planktonic foraminiferal species biogeography, the resulting distribution patterns are discussed and compared to those available from the literature.

## Results

### Patristic Distances

**Figure 1:**
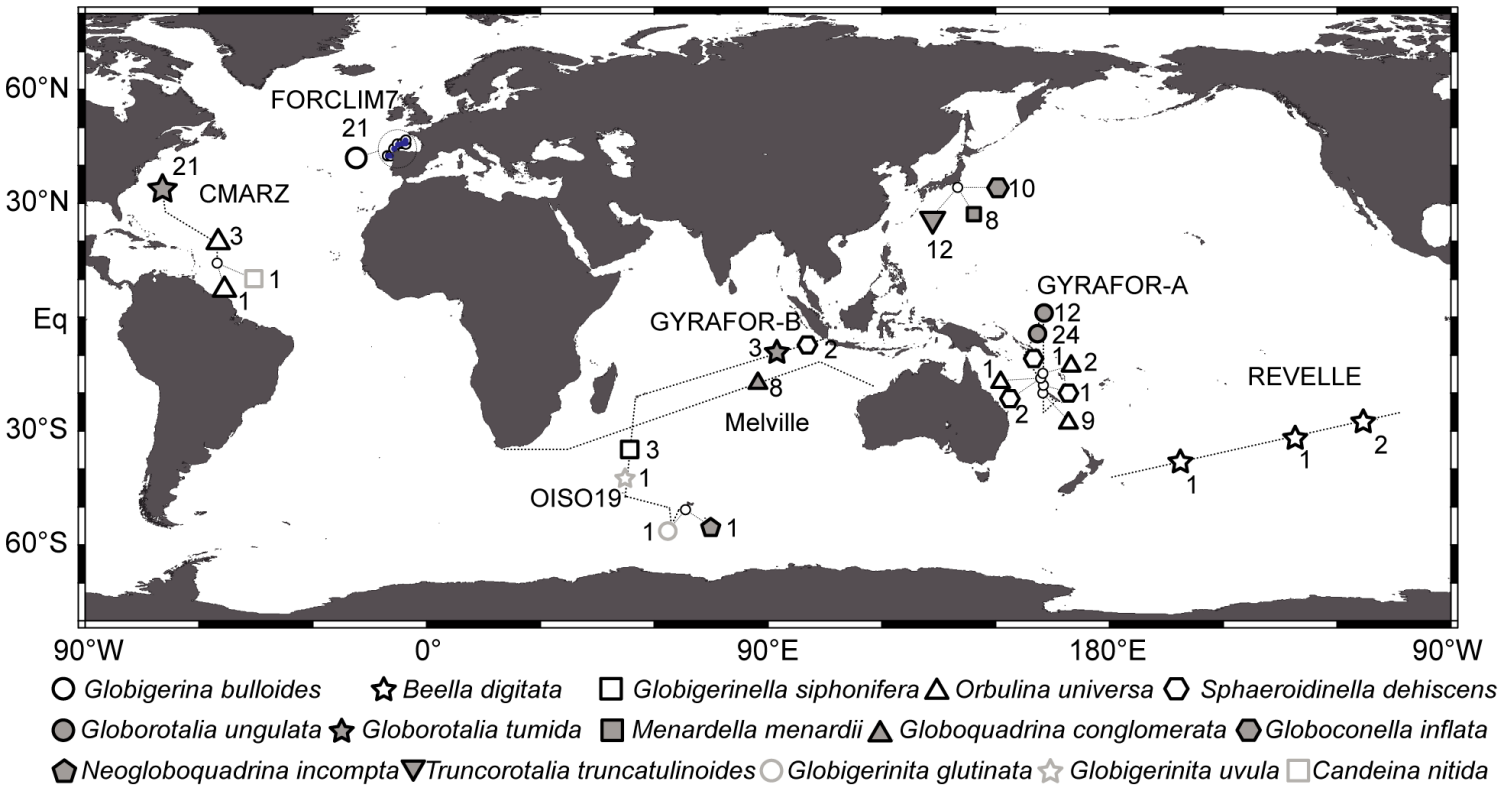
Sample map of newly assembled data. Geographic location and labels of the oceanic stations sampled for acquiring new SSU rDNA sequences of individual planktonic foraminifera. Numbers next to labels correspond to the number of sequences obtained for each station. Dashed lines represent ship routes of cruises CMARZ (2006), FORCLIM7 (2009), GYRAFOR-A (2008), GYRAFOR-B (2007), OISO19 (2011) and REVELLE (2000).

Accounting for taxonomic synonymies and ambiguous assignations (see Material and Methods), we retained 1217 SSU rDNA sequences from NCBI. Adding the 135 newly obtained sequences from living foraminifera collected in the world oceans (Fig. 1), we finally compiled a global dataset of 1352 sequences belonging to 25 morpho-species.

**Figure 2:**
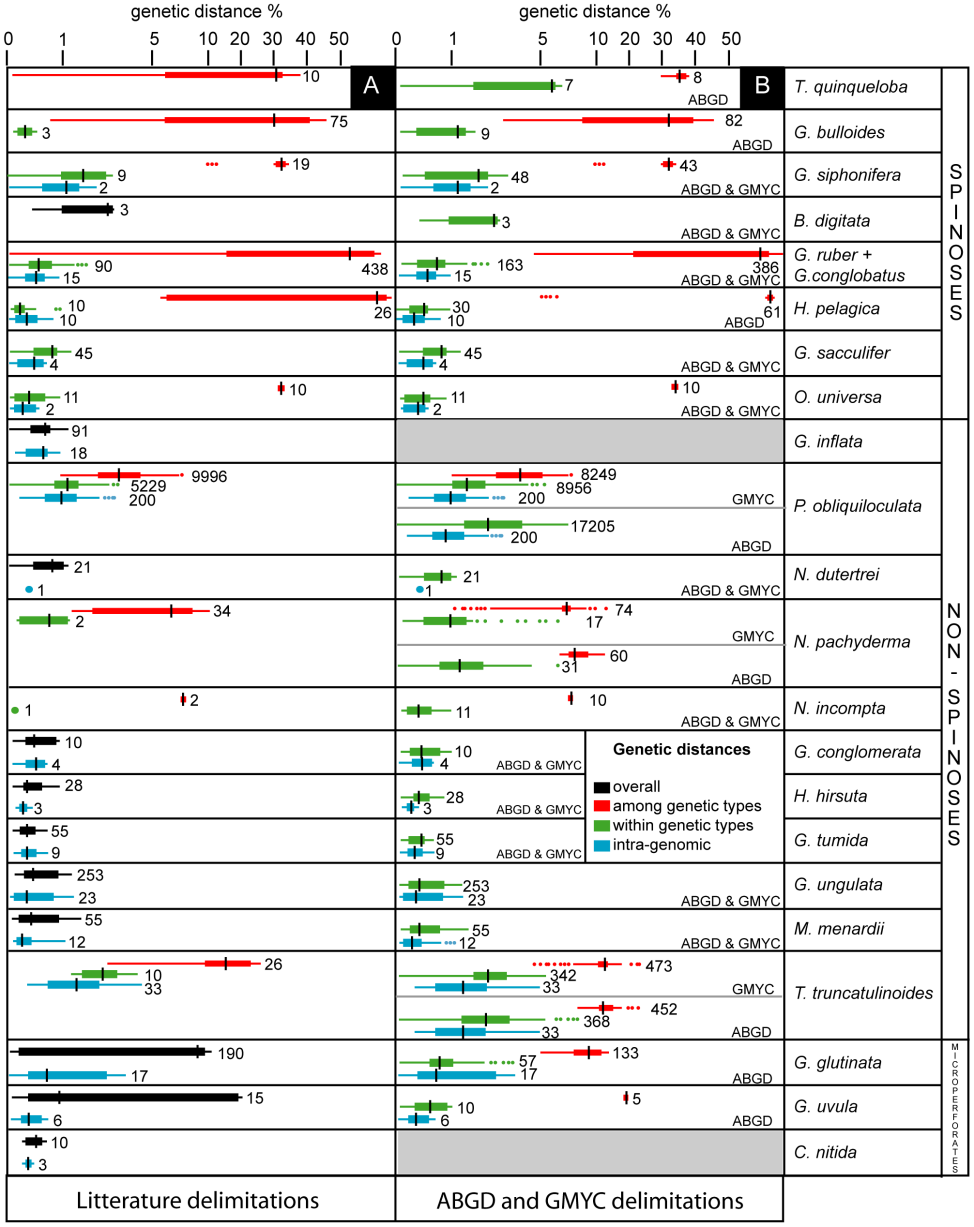
Patristic distances within and among genetic types of planktonic foraminifera. Boxplot distribution of SSU genetic diversity (patristic distances, expressed as percent of nucleotide change) within each studied planktonic foraminiferal morpho-species. Central box represents the upper and lower quartiles; whiskers represent the extreme of the data with points exceeding Q3+1.5IQ or below Q1-1.5IQ (Q1: 1^st^ quartile, Q3: 3^rd^ quartile and IQ: Q3-Q1) considered as outliers; the central mark gives position of the median; numbers indicate the number of pairwise distances included in the distribution. Red boxes correspond to inter-genetic type distances, green boxes to intra-genetic type distances, blue boxes to intra-genomic distances and black boxes to overall genetic distances. A: Boxplot distribution using species delimitations and specimens identifications according to NBCI database and literature; B: boxplot distribution using species delimitations and specimens identifications according to ABGD and GMYC analyses.

Due to lengths heterogeneities of the NCBI sequences, only 714 sequences (corresponding to 395 non-identical sequences) of a homologous block of ∼700 bp were used for calculations of patristic distances. As a result of procedure requirement, inter- and intra-type distances could be estimated for 9 out of 25 morpho-species (Fig. 2A). Inter-type distances are highly variable between morpho-species, their medians ranging from 3.5% (*Turborotalia quinqueloba*) to 64% (*Hastigerina pelagica*). Intra-type distances are less variable than inter-type distances as their medians range from 0.21% (*H. pelagica*) to 3.2% (*Truncorotalia truncatulinoides*). Overall intra-type distances are also significantly lower than inter-type distances (Table S1). For *Globigerinella siphonifera*, *Orbulina universa*, *H. pelagica* and *Neogloboquadrina incompta*, we observe a clear distance gap between inter- and intra-type distances, then implying that genetic types for these 4 morpho-species correspond to genuine cryptic species. The case of *Neogloboquadrina pachyderma* remains ambiguous as the distance gap is almost nil. The very low diversity occurring between some of the previously defined genetic types of *Globigerina bulloides* and *T. quinqueloba* (0.90% and 0.12%, respectively), suggests that these morpho-species may have been oversplit. For 3 morpho-species (*Globigerinoides ruber+conglobatus*, *Pulleniatina obliquiloculata* and *T. truncatulinoides*), intra- and inter-type distances overlap, suggesting that at least some of their previously defined genetic types within may not correspond to genuine species. Because overall and intra-genomic distances are within the same range, the absence of cryptic diversity is confirmed in *Globigerinoides sacculifer* [22].

Overall, intra-genomic distances have been estimated for 17 morpho-species. Their medians range from 0.25% (*Menardella menardii*) to 2.6% (*T. truncatulinoides*). These distances are thus of the same range, or lower, than intra-type distances within the same morpho-species (Table S1). In 11 morpho-species, neither inter-type nor intra-type distances have been estimated. The sampled specimens of *Globoquadrina conglomerata*, *Hirsutella hirsuta*, *Globorotalia tumida*, *Globorotalia ungulata*, *M. menardii* and *Candeina nitida* do not hide cryptic diversity given that their total genetic distances are reduced and are of the same range as the intra-genomic distances (Table S1). Likewise, the analyzed sequences of *Globoconella inflata* correspond to a single species but we note that due to their short length, sequences of the second described genetic type [21] were all excluded from the dataset. Overall distances within *Neogloboquadrina dutertrei* and *Beella digitata* seem to match intra-species distances but data are too scarce to draw firm conclusions. Conversely, these distances are significantly higher that the intra-genomic distances in *Globigerinita uvula* and *Globigerinita glutinata*, suggesting that they may constitute complexes of yet unknown cryptic species. The case of these two latter morpho-species points to a major drawback of the patristic distance approach: it requires a-priori delimitations.

### Automatic genetic type delimitation methods

#### Automatic Barcode Gap Discovery (ABGD)

ABGD sorts the sequences into hypothetical species based on the barcode gap that can be observed whenever the divergence among individuals belonging to the same species is smaller than divergence among individuals from different species. Since ABGD does not require any prior attribution of taxonomic units, the sequences that lacked genetic type labels in the initial dataset were included in the analysis, which is then run on the same SSU region used for patristic distances calculation (462 non-identical sequences of ∼700 bp; 23 morpho-species).

No barcode gap was identified for 7 morpho-species (Globigerinoides sacculifer, Beella digitata, Globorotalia tumida, Hirsutella hirsuta, Neogloboquadrina dutertrei, Pulleniatina obliquiloculata and Globoconella inflata). Based on this method, the sampled material of each of these morpho-species corresponds to a single genuine species (Table 1). In G. inflata, however, we cannot exclude that our dataset contains only sequences from one of the two types identified by [21]. For 6 morpho-species (Orbulina universa, Turborotalia quinqueloba, Truncorotalia truncatulinoides, Neogloboquadrina pachyderma, Neogloboquadrina incompta, and Globigerinita uvula), ABGD identifies only one species delimitation optimum (i.e., one MOTUs richness plateau). For the remaining 8 morpho-species, it identifies several species delimitation optima. As other delimitation optima lead to mis-assignment of clones from the same individual into distinct MOTUs, we retain the one-MOTU delimitation in Globorotalia ungulata, Menardella menardii and Globoquadrina conglomerata, and the four-MOTUs delimitation in Globigerinita glutinata. For the remaining 4 morpho-species, we consider the first MOTUs plateau (i.e. the plateau corresponding to the maximum number of species) for putative species delimitation. The first MOTUs plateau is usually considered as corresponding to the putative species delimitation [42], [44]. For verification, in Globigerina bulloides, Globigerinoides ruber+conglobatus Globigerinella siphonifera and Hastigerina pelagica, we also calculated patristic distances for the genetic type delimitations corresponding to the second MOTUs plateaus generated by ABGD (File S1). We obtain high intra-specific distances (up to 10%) corresponding to 4 to 8 times the intra-genomic distances, confirming that the second MOTUs plateaus are less likely to correspond to genuine species delimitations. As a consequence, we find that ABGD qualifies 7 putative species within G. bulloides, 5 within G. ruber+conglobatus, and 3 within G. siphonifera and H. pelagica.

**Table 1:** Species delimitation according to the literature, literature delimitation checked by patristic distances, ABGD species delimitations and GMYC species delimitations.

morphospecies	literature	patristic	ABGD	GMYC
*T. quinqueloba*		invalidated (1)		
	Ia		I	*T. quinqueloba* *
	Ib		I	*T. quinqueloba* *
	IIa		II	*T. quinqueloba* *
	IIb		II	*T. quinqueloba* *
	IIc		II	*T. quinqueloba* *
	IId		n. a.	*T. quinqueloba* *
*G. bulloides*		invalidated (1)		
	Ia		Ia	*G. bulloides* *
	Ib		Ib-c-e	*G. bulloides* *
	Ic		Ib-c-e	*G. bulloides* *
	Ie		Ib-c-e	*G. bulloides* *
	Id		Id	*G. bulloides* *
	IIa		IIa	*G. bulloides* *
	IIb		IIb-d-f	*G. bulloides* *
	IId		IIb-d-f	*G. bulloides* *
	IIf		IIb-d-f	*G. bulloides* *
	IIe		IIe	*G. bulloides* *
	IIc		IIc	*G. bulloides* *
*B. digitata*	n. d.	*B. digitata*	*B. digitata*	*B. digitata*
*S. dehiscens*	n. d.	n. a.	n. a.	*S. dehiscens*
*G. siphonifera*		validated		
	I		I	I
	IIa = II		II	II
	IIb = III		III	III
*G. ruber + conglobatus*		invalidated (2)		
	*G. conglobatus*		*G. conglobatus*	*G. conglobatus*
	IIa		IIa	IIa
	IIa1		IIa	IIa
	IIa2		IIa	IIa
	IIb		n. a.	IIb
	Ia		Ia	Ia
	Ib1		Ib	Ib
	Ib2		Ib	Ib
	pink		pink	pink
*G. sacculifer*	*G. sacculifer*	validated	*G. sacculifer*	*G. sacculifer*
*O. universa*		validated		
	II	n. a.	n. a.	II
	I		I	I
	III		III	IIIa = III
	III	n. a.	n. a.	IIIb = III
*H. pelagica*		validated		
	I		I	n. a.
	IIa		IIa	n. a.
	IIb		IIb	n. a.
*T. truncatulinoides*		invalidated (2)		
	I		I-II	I
	II		I-II	II
	III		III-IV	III-IV
	IV		III-IV	III-IV
	V		V	V
*H. hirsuta*	n. d.	*H. hirsuta*	*H. hirsuta*	*H. hirsuta*
*G. conglomerata*	n. d.	*G. conglomerata*	*G. conglomerata*	*G. conglomerata*
*M. menardii*	n. d.	*M. menardii*	*M. menardii*	*M. menardii*
*G. ungulata*	n. d.	*G. ungulata*	*G. ungulata*	*G. ungulata*
*G. tumida*	n. d.	*G. tumida*	*G. tumida*	*G. tumida*
*N. pachyderma*		validated		
	I		I	I
	II		II-III-V-VI	II-III-VI
	III		II-III-V-VI	II-III-VI
	VI		II-III-V-VI	II-III-VI
	V		II-III-V-VI	V
	IV		IV	IV
	VII		VII	VIIa
	VII		VII	VIIb
*N. incompta*		validated		
	I		I	I
	II		II	II
*N. dutertrei*	*N. dutertrei*	inconclusive	*N. dutertrei*	*N. dutertrei*
*G. inflata*		inconclusive		
	I		I	I
	II		n.a.	II
*P. obliquiloculata*		invalidated (2)		
	I		*P. obliquiloculata*	I
	IIa		*P. obliquiloculata*	II
	IIb		*P. obliquiloculata*	II
*G. uvula*	n. d.	inconclusive		
			I	*G. uvula* *
			II	*G. uvula* *
*G. glutinata*	n. d.	inconclusive		
			I	*G. glutinata* *
			II	*G. glutinata* *
			III	*G. glutinata* *
			IV	*G. glutinata* *
*C. nitida*	n. d.	*C. nitida*	inconclusive	*C. nitida* *

(1): delimitation invalidation due to improbable inter-species distances; (2): delimitation invalidation due to overlapping of intra- and inter-species distances; n.d.: no genetic type defined in the literature; n.a.: genetic type not included in the dataset;

*: non-significant likelihood ratio test.

ABGD putative species delimitations are similar to previously published genetic types in 5 morpho-species: *O. universa*, *G. sacculifer*, *G. siphonifera*, *H. pelagica* and *N. incompta* (Table 1). The method lumps some of the previously defined genetic types into the same species in 7 morpho-species: *T. quinqueloba*, *G. bulloides, G. ruber+conglobatus*, *N. pachyderma*, *P. obliquiloculata*, *G. inflata* and *T. truncatulinoides*. Putative species delimitations are here obtained for the first time in 9 morpho-species: *B. digitata*, *N. dutertrei, G. tumida*, *H. hirsuta*, *G. ungulata*, *G. menardii*, *G. conglomerata, G. glutinata* and *G. uvula*. We calculated patristic distances among and within putative species as they are defined by the ABGD method. As expected for a distance-based method [42], such calculations lead to non-overlapping distributions of intra- and inter-genetic types distances (Fig. 2B), except for *T. truncatulinoides*, which still exhibits a slight overlap. In *N. pachyderma*, ABGD favors an alternative genetic type delimitation that is clearly cross-validated when processed through a second patristic distances approach. Although the gap between intra- and inter-type distances is extremely reduced, published genetic types are also validated and we consequently cannot determine which delimitation (i.e., literature or ABGD) should be favored.

#### General Mixed Yule Coalescent (GMYC)

GMYC is a likelihood method for delimiting species by fitting intra- and inter-species branching models to reconstruct molecular trees. Since it does not require the use of sequences of the same length, 649 non-identical sequences belonging to 24 morpho-species were equally analyzed. To avoid long calculation time and ambiguous alignments, we divided the dataset into 5 subsets of sequences, each of them incorporating sequences belonging to 3 to 7 morpho-species following [17].

**Figure 3:**
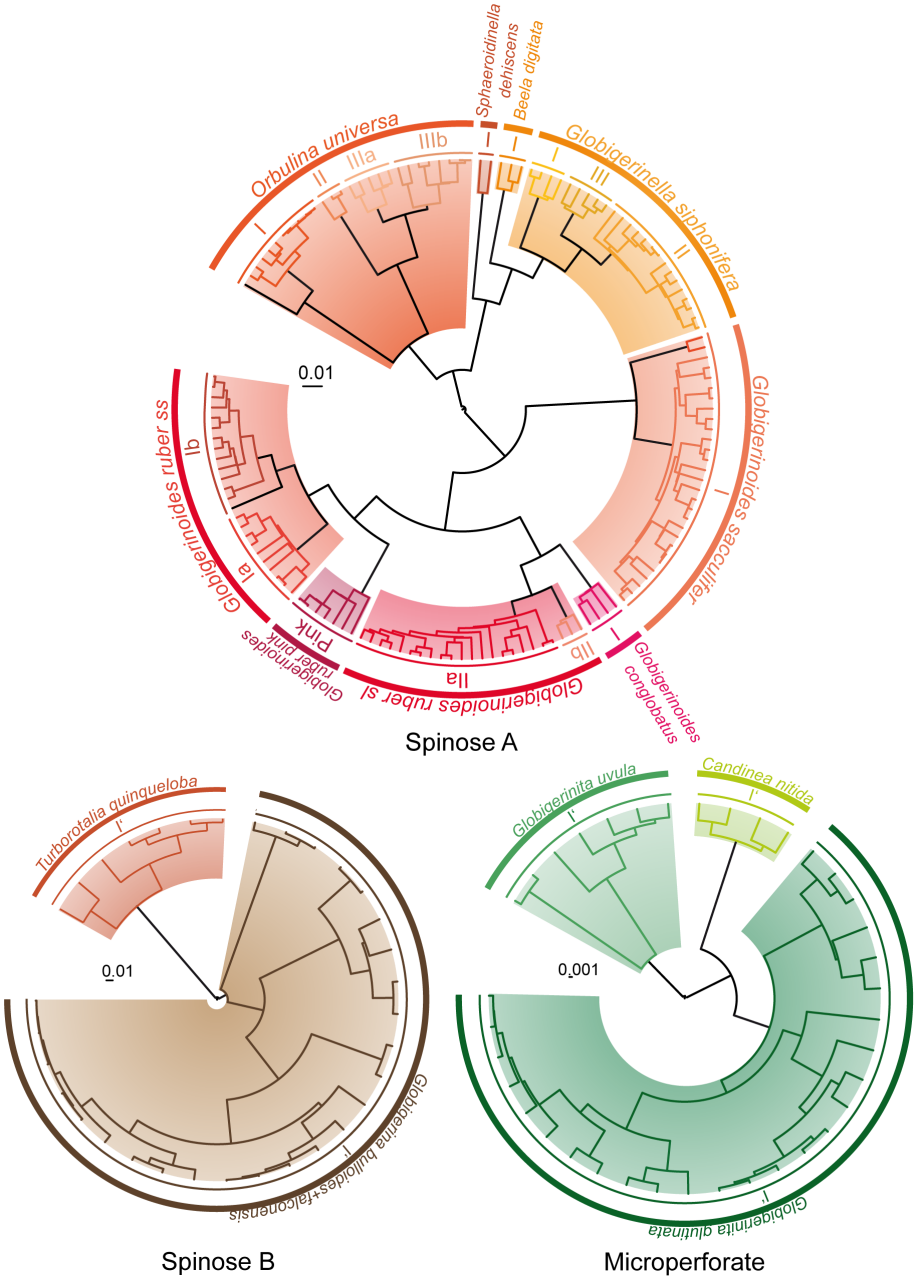
Ultrametric trees “Spinose A”, “Spinose B” and “Microperforate”with GMYC delimitations. Delimitations are significant only for “Spinose A” (see Table 2). Colored branches correspond to GMYC clusters and outer circles correspond to the names of the morpho-species (outer arc) and plausible biological species (inner arc) (see Table 1). Symbols associated to specific colors indicate clones sequenced from the same individuals. For sequences accession numbers see Figure S1.

**Figure 4:**
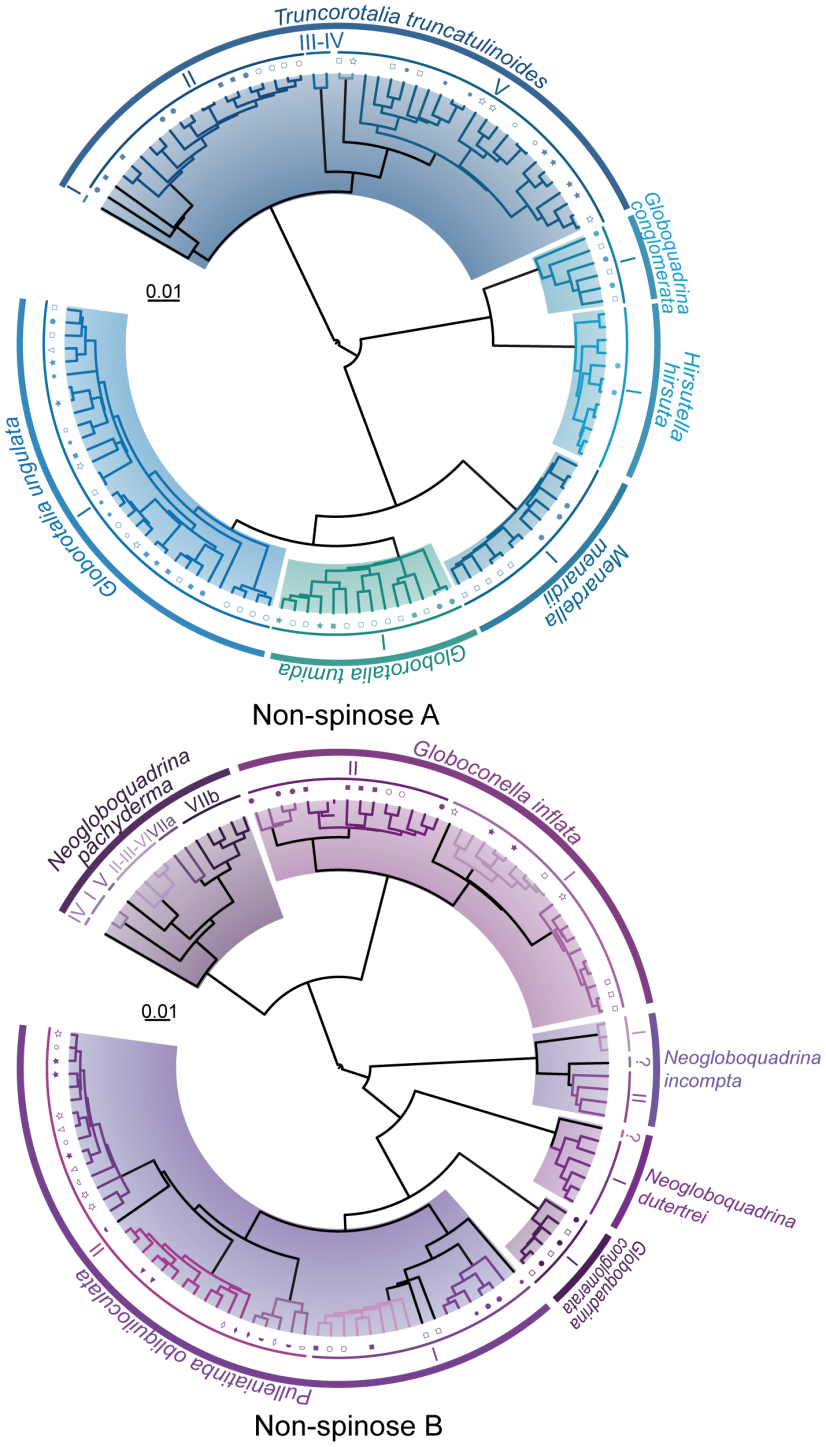
Ultrametric trees “Non-spinose A” and “Non -spinose B” with GMYC delimitations. Delimitations are significant for both trees (see Table 2). Colored branches correspond to GMYC clusters and outer circles correspond to the names of the morpho-species (outer arc) and plausible species (inner arc) (see Table 1). Symbols associated to specific colors indicate clones sequenced from the same individuals. For sequences accession numbers see Figure S1.

For 3 ultrametric trees (“Spinose A”, “Non-spinose A” and “Non-spinose B”; Figs. 3 and 4; Table 2; Figure S1), the likelihood of the GMYC model is significantly higher than that of the null model and thus supports the hypothesis that those trees include several biological species. The GMYC method applied to the tree “Spinose A” lead to an estimate of 19 putative species, 2 of which containing a single individual (Fig. 3). Isolated sequences AJ229109 (*Orbulina universa*), Z839665 (*Globigerinoides ruber*), JQ004126 and Z83964 (*Globigerinoides sacculifer*) (Figure S1) correspond to old (submitted to the NCBI database back in 1996) or short sequences (<500 bp). Furthermore, most substitutions of these isolated sequences are concentrated on the 5′ end region. We thus speculate that the apparent divergence of these sequences is likely to be the result of PCR and/or sequencing artifacts that were more frequent with older amplification and sequencing techniques. Furthermore, patristic distances between these divergent sequences within *G. ruber* and *G. sacculifer* and the other sequences from their closest putative species were clearly of intra-type distance range, confirming that the corresponding sequences should not be considered as separate species (File S1). Consequently, we do not retain these isolated sequences as genuine species. Sequences of the previously defined Type III of *O. universa* [30] are separated into two clusters corresponding to long (∼1000 bp) sequences and to short (<450 bp) and old sequences, respectively. This GMYC classification is also a likely artifact as it only reflects differences in length and quality of the sequences. Unfortunately, the patristic distance approach could not be applied on the Type IIIb of *O. universa* as the sequences were too short. Altogether, interpretation of the genetic type delimitations by the GMYC method reduces the number of putative species to 3 in *O. universa*, 6 in *G. ruber+conglobatus*, and 1 in *G. sacculifer* (Table 1). In *G. ruber+conglobatus*, Types IIa1 and IIa2 [45] and Types Ib1 and Ib2 [9] are clustered into single MOTU here renamed Type IIa and Type Ib, respectively. In *Globigerinella siphonifera*, GMYC delimitates 3 putative species corresponding to the previously defined genetic types [9]. Finally, it delimitates one cluster for each of the newly sequenced morpho-species *Sphaeroidinella dehiscens* and *Beella digitata* (Fig. 3).

**Table 2:** Characteristics of the ultrametric trees used for GMYC species delimitation.

Tree name	LR test	sequences nb	seq/sp	sequences sizes (bp)	Morphospecies
Spinose A	***	143	9.5	196–1059, 772	*G. sacculifer, O. universa, S. dehiscens, G. siphonifera, B. digitata, G. ruber, G. conglobatus*
Spinose B	ns	33	1.83	323–1169, 882	*G. bulloides, G. falconensis, T. quinqueloba*
Macro A	*	123	12.3	1021–3592, 1173	*T. truncatulinoides, H. hirsuta, G. conglomerata, M. menardii, G. ungulata, G. tumida*
Macro B	**	121	8.1	469–3412, 1028	*N. pachyderma, N. incompta, N. dutertrei, P. obliquiloculata, G. inflata, G. conglomerata*
Micro	ns	37	ns	436–1022, 922	*G. uvula, C. nitida, G. glutinata*

LR test: likelihood ratio test of the GMYC delimitation, n.s =  non significant;

*,**,*** =  significant. seq/sp: mean number of sequences per morphospecies, size of the sequences as min-max, mean.

The GMYC method applied to the tree “Non-spinose A” lead to an estimate of 12 putative species, 3 of which containing a single individual (Fig. 4). It delimitates 7 putative species within *Truncorotalia truncatulinoides* but clusters the previously defined Types III and IV [16], [20] as a single putative species. We note that four clones of *T. truncatulinoides* (KJ633252, KJ633259, FJ643341 and FJ343329; Figure S1) do not cluster into the same species than other clones of the same individual. As we chose to assign clones from the same individuals to the same putative species, the number of putative species within *T. truncatulinoides* is then reduced to four (Fig. 4). These GMYC-delimitated genetic types lead to overlapping intra- and inter-type patristic distances but we note that such overlapping distances are much reduced compared to those calculated from the literature-based patristic approach. Furthermore, GMYC analyses suggest that *Hirsutella hirsuta, Globorotalia tumida, Globorotalia ungulata, Menardella menardii* and *Globoquadrina conglomerata* do not contain cryptic diversity.

The GMYC method applied to the tree “Non-spinose B” leads to an estimate of 25 putative species, 8 of which based on a single individual (Fig. 4). Six putative species are delimitated within *Neogloboquadrina pachyderma*. While Types II, III and VI [34] of *N. pachyderma* are clustered into a single putative species and two sequences without former genetic type assignation are identified as a new putative species (Type VIIb), these delimitations lead to overlapping intra- and inter-type patristic distances. The topology of the tree is highly sensitive to model and/or method changes, possibly because only one sequence is available for 5 out of the 7 genetic types defined by [34]. Given that *N. pachyderma* phylogram (PHYML) and ultrametric tree (BEAST) recover quite different relationships (File S1), patristic distances were not pertinent for testing GMYC delimitations. In *Neogloboquadrina incompta*, application of the GMYC method delimitates 2 putative species corresponding to the previously defined genetic types of [46], and isolates the sequence AY453130 as a possible third species. It also suggests that there are 2 putative species in *Neogloboquadrina dutertrei*, one of which containing a single individual (AY241708). The species status of the two isolated and short sequences from *N. incompta* and *N. dutertrei* could not be tested through the patristic distance approach. According to the GMYC species delimitation, the sampled specimens of *Globoquadrina conglomerata* do not contain cryptic diversity. GMYC delimitates 4 clusters and one isolated sequence within *Globoconella inflata* but clones from the same individual appear to cluster into different putative genetic types. After grouping these clones into their respective species, the number of putative species within *G. inflata* is reduced to two. Again, due to the short length of the sequences, we could not apply the patristic distances approach to the Type II of *G. inflata*. Likewise, the 5 clusters and 4 isolated sequences delimited within *Pulleniatina obliquiloculata* are clustered into two putative species after grouping clones into the same species. *Pulleniatina obliquiloculata* GMYC-delimitated and genetic types defined in the literature lead to overlapping intra- and inter-type patristic distances (Fig. 2). Even after close examination of the dataset of *P. obliquiloculata*, we were not able to infer a possible artifact that might explain why species delimitation is unsatisfactory within this morpho-species.

Last, likelihoods of the GMYC model are not significantly higher than that of the null model for the trees “Spinose B” and “Microperforate” (Fig. 3; Table 2). Validation of the null model would imply that each of those trees would correspond to a single genetic type, which appears highly unrealistic as it would cluster very distinct morpho-species such as *Globigerina bulloides* and *Globigerina falconensis* or *Globigerinata glutinata* and *Globigerinita uvula*. Therefore these negative results clearly point to a lack of statistical power of the GMYC method in those still poorly-sampled organisms.

## Discussion

### Heterogeneity of previous genetic type delimitations

Our study shows that the empirical delimitations available in the literature suffer from subjectivity although the heterogeneity of the dataset complicates their review using more objective criteria. Those delimitations are apparently not homogenous: some previously-defined genetic types are likely to correspond to valid taxonomic units whereas others probably represent sub-species or population level structuring. Among the morpho-species for which genetic type delimitation seems accurate, intra-specific distances are quite variable as well as the range of the distance gap between intra- and inter-specific distances (Fig. 2). This phenomenon may be the result of the heterogeneity of substitution rates observed between planktonic foraminiferal lineages and even sometimes between more closely related morpho-species [13]. Consequently, as already observed in other cases (e.g. [47]), the design of a unique threshold value between intra- and inter-specific distances may not be appropriate in the case of planktonic foraminifera due to strongly heterogeneous evolutionary rates among clades. This challenges the possibility of a straightforward use of our results on other protists groups, all the more when these groups lack a robust phylogenetic morpho-taxonomy as it is often the case [48].

### ABGD: an efficient method to build species delimitation hypotheses in planktonic foraminifera

With the exception of *Truncorotalia truncatulinoides* and as expected for a distance-based method, delimitations resulting from ABGD are validated when they are processed through a second patristic distance comparaison since they lead to non-overlapping distributions of intra- and inter-genetic type distances (Fig. 2, Table 1). This second patristic distance approach run on ABGD delimitations also comforts the first MOTUs plateau as the optimal genetic type delimitation, as also shown other groups of organisms [42], [44]. Even if the ABGD and patristic distance approaches are highly congruent, calculation of patristic distances is still of interest as it comforts genuine species delimitations by screening unrealistic hypotheses.

Running ABGD is fast and efficient enough for building taxonomic hypotheses, even when only a few sequences are available (e.g., *Globigerinita uvula*). Consequently, this method should be intended first for generating planktonic foraminiferal species delimitation hypotheses. Importantly, a current limit of this method is the requirement that the alignment is void of missing data. A direct consequence is the exclusion of potential cryptic species from analyses (e.g., Types II and IIIb of *Orbulina universa*, Type IIb of *Globigerinoides ruber* and Type II of *Globoconella inflata*). Thus, the generalized use of ABGD for planktonic foraminiferal species delimitation, and thus the development of a DNA barcode, would require a standardization of the length and covered regions of the sequenced and deposited SSU regions.

### GMYC: a distance-independent method for building species delimitations in planktonic foraminifera

In spinose planktonic foraminifera, GMYC isolates numerous divergent sequences as putative species (Fig. 3). These isolated sequences rarely correspond to genuine species but usually result from PCR and/or sequencing artifacts that were more frequent with older amplification and sequencing techniques. These cases show that sequence divergence may be an accurate measure of speciation only if the quality of sequencing is reasonably well-controled. In a few cases, the GMYC method assigns SSU copies from the same individuals to different putative species. This assignment may be the result of 3 phenomena: (i) non-concerted evolution between copies within the genome [49], whose extant remains unfortunately largely unknown in foraminifera [50] and may be extremely variable between species [51]; (ii) PCR and/or sequencing errors can produce enough noise when genetic distances are low, obliterating the true phylogenetic signal [52]; and (iii) horizontal gene transfer, a phenomenon rare in eukaryotes taxa and never shown for rDNA [53]. As the intra-genomic diversity is extensive within *Pulleniatina obliquiloculata*, we speculate that non-concerted evolution of SSU copies within genomes may have generated the widespread sorting of clones from the same individuals into different clusters observed for this morpho-species (tree “Non-spinose B”; Fig. 4). Likewise, a slight non-concerted evolution of the SSU copies may be also involved in the case of the four isolated clones of *Truncorotalia truncatulinodes* (KJ633259, KJ633252, FJ643341 and FJ643329). Genetic diversity within *Globoconella inflata* was mostly studied on the basis of the fast-evolving ITS genes because SSU divergence is very low [21]. A few PCR and/or sequencing artifacts or a slightly non-concerted evolution of SSU rDNA copies might have induced the assignment of clones to different putative species. These examples illustrate the limits of the use of ribosomal genes sequences for species delimitation. Yet, even in the cases of protists for which genetic studies are more advanced, these multi-copy genetic markers are often preferred over single-copy markers like COI which have their own shortcomings [54].

Together, GMYC delimitations corroborate all previous genetic type delimitations supported as genuine species using the patristic distance approach, with the exception of *Neogloboquadrina pachyderma* (Table 1). For all morpho-species but *Truncorotalia truncatulinoides*, *N. pachyderma* and *Pulleniatina obliquiloculata*, the new putative species delimitations obtained through GMYC are supported as genuine species by the patristic distance approach.

Our study shows that GMYC is an efficient genetic distance-independent method for planktonic foraminiferal species delimitation using SSU rDNA sequences. However, results also demonstrate that GMYC-based delimitations can be heavily impacted by data quality and thus requires thorough inspection. Because the sequence overlap/missing data criteria are less critical than in distance-based methods, the GMYC analysis identifies more putative species than ABGD and patristic distances analyses (Figs. 3 and 4; Table 1). While GMYC proves useful, we also recover very doubtful results where the GMYC model could not be favored over the null model of a single coalescent population (i.e., trees “Spinose B” and “Microperforate”; Figs. 3 and 4, Table 2 and Figure S1). Comparison of these cases suggests guidelines on how to design sequence datasets for GMYC analysis. We note that in our study, the statistically significant GMYC species delimitations tend to be associated with: (i) large datasets involving more than 3 morpho-species; (ii) datasets with few ambiguously aligned regions, i.e., datasets including closely related morpho-species; and (iii) datasets with overall homogenous sequence length. Indeed, the reduced size of the dataset might explain failure to reject the null model for “Spinose B” and “Microperforate” species delimitations and calls for more sequences from a larger set of morpho-species. In the case of the tree “Spinose B”, integration of the divergent morpho-species *Turborotalia quinqueloba* might also be at stake in putative species delimitation failure. Finally, the GMYC-delimitated clusters within the Type III of *O. universa* (Fig. 3), which apparently does not correspond to a distinct genuine species, show that sequence length heterogeneity may strongly influence GMYC-based species delimitation.

### Towards a molecular taxonomy of planktonic foraminifera

The patristic distance, ABGD and GMYC analyses gave us the opportunity to confirm delimitations, and to establish new hypotheses for invalidated cases. Indeed, we show that only 6 out of the 12 morpho-species in which cryptic diversity was characterized in the literature had all their genetic types corresponding to genuine species (Table 1). Conversely, whatever method we used, about half of the genetic types identified so far in the literature appear unlikely to represent genuine biological species. This overall tendency to over-split may result from the fact that distance thresholds as well as more sophisticated concepts such as compensatory base change (e.g. [54]) were seldom discussed in the case of planktonic foraminifera, leading to potential over-interpretation of phylogenetic trees.

Even if the ABGD and GMYC putative species delimitation methods are independent, their results appear highly congruent: within the SSU rDNA sequences considered by both approaches, the ABGD method delimitates 29 genetic types, 25 (86%) of which being identical to those defined on the basis of the GMYC method (Table 1). These results expand previous reports suggesting a strong agreement between distance-based and GYMC delimitations [55]. Among putative species, those which are identically and independently defined by both methods most probably correspond to genuine cryptic or pseudo-cryptic biological species. The high congruence between the two methods also suggests that the genetic types delimitated using a single approach are plausible hypotheses. Our data confirm that GMYC, when compared to distance methods, seems to over-split groups [55], [56], [57], whereas ABGD tends to over-lump previously defined or GMYC-defined genetic types (e.g., *Neogloboquadrina pachyderma* in Table 1).

Finally, based on the available data, putative species delimitations remain ambiguous only for *N. pachyderma*, *Truncorotalia truncatulinoides* and *Pulleniatina obliquiloculata*. In *N. pachyderma*, GMYC delimitations are clearly invalidated by the patristic distance approach but ABGD and literature delimitations are both plausible (Fig. 2). This ambiguity probably results from the unresolved phylogenetic relationships occurring between the possible genetic types, which result themselves from the small number of sequences available for this genetically highly diverse morpho-species (Table 3). All delimitation hypotheses for *T. truncatulinoides* are invalidated through the patristic distance approach. Exploring cryptic genetic diversity in *T. truncatulinoides* suffers a lack of molecular homogeneity, since the Types I, II, III and IV have been characterized based on ITS rDNA sequences [20], whereas the Type V was characterized based on SSU rDNA ones [16]. Only one SSU rDNA sequence has been deposited in NCBI for the Types I, III and IV of *T. truncatulinoides* and the resolving power of the GYMC and ABGD methods for species delimitation is therefore very low [42]. In particular, ABGD and GMYC are not congruent on the taxonomic status of the closely related Types I and II (Table 1). Using ITS rDNA sequences, [20] suggested genetic isolation between these two Types that may be explained by a geologically recent (100 to 200 kyrs) speciation event. Consequently, recent speciation events may not be detected by both methods, and the resulting conflicting species delimitations originating from ABGD and GMYC highlight that further sequencing of the faster-evolving ITS rDNA marker is needed for investigation of possible recent speciation events. Literature and GMYC genetic type delimitations in *P. obliquiloculata* are clearly invalidated through the patristic distance approach. Although application of ABGD lumps all available sequences into a single MOTU, its specific status remains unclear on the basis of patristic distances. On the one hand, distance values within *P. obliquiloculata* are high (up to 7.6%), suggesting that this morpho-species may be a complex of several cryptic species but, on the other hand, as the intra-genomic distances are also particularly high (up to 3.3%), we cannot exclude that it may not hide any cryptic diversity. The lack of barcode gap within *P. obliquiloculata* either suggests that the potential speciation events, if any, have occurred very recently [42], [43] and/or that it is not detected due to a high intra-genomic diversity. Likewise, heterogeneous evolutionary rates between (and also possibly within) the genetic types of *P. obliquiloculata* may generate similar values for intra-genetic type distances within fast evolving types and for inter-genetic type distances.

**Table 3:** Number of sequences analyzed in the different datasets.

Morphospecies	NCBI+new	Patristic literature			ABGD	GMYC
		inter	intra	intraG		
*T. quinqueloba*	8	6	0	0	8	7
*G. bulloides*	85	13	8	0	14	23
*G. siphonifera*	52	8	7	4	14	24
*B. digitata*	4	3*	0	0	3	3
*S. dehiscens*	6	0	0	0	0	2
*G. sacculifer*	114	-	10	8	10	29
*O. universa*	50	7	7	4	7	27
*G. ruber+conglobatus*	313	33	31	21	36	58
*H. pelagica*	145	9	9	12	14	-
*T. truncatulinoides*	51	9	5	28	41	46
*H. hirsuta*	72	8*	0	6	8	12
*G. tumida*	18	11*	0	11	11	14
*G. ungulata*	37	23*	0	21	23	31
*M. menardii*	14	11*	0	10	11	14
*G. conglomerata*	32	5*	0	5	5	6
*G. inflata*	70	14	0	11	14	48
*P. obliquiloculata*	258	175	175	164	181	239
*N. dutertrei*	10	7	0	1	7	8
*N. pachyderma*	17	9	4	0	14	15
*N. incompta*	13	3	2	0	7	8
*G. glutinata*	38	24*	0	17	24	24
*C. nitida*	7	4*	0	0	4	4
*G. uvula*	7	6*	0	4	6	7

NCBI+new: number of sequences available on NCBI and number of new sequences added. Patristic literature: number of sequences included in the dataset based on literature species definitions; inter, intra, intraG: number of sequences used for inter-genetic type, intra-genetic type and intra-genomic distances calculations, respectively.

*: number of sequences used for total distances calculation (for the morpho-species for which no cryptic species have been identified). ABGD: number of sequences included in the dataset used for ABGD species delimitation. GMYC: number of sequences included in the dataset used for GMYC species delimitation.

### Integrative taxonomy and biogeography of planktonic foraminiferal cryptic species

The new or confirmed delimitations discussed above are based on molecular data which have their own limitations, in particular when dealing with closely-related and young species [58]. Below and in Text S1, in an attempt to review the species status of modern planktonic foraminifera based on an integrative taxonomy [59], we compile our data with previously published non-molecular evidences, including biogeographic, ecological and/or morphological differentiations that can occur among (pseudo)cryptic species.

**Figure 5:**
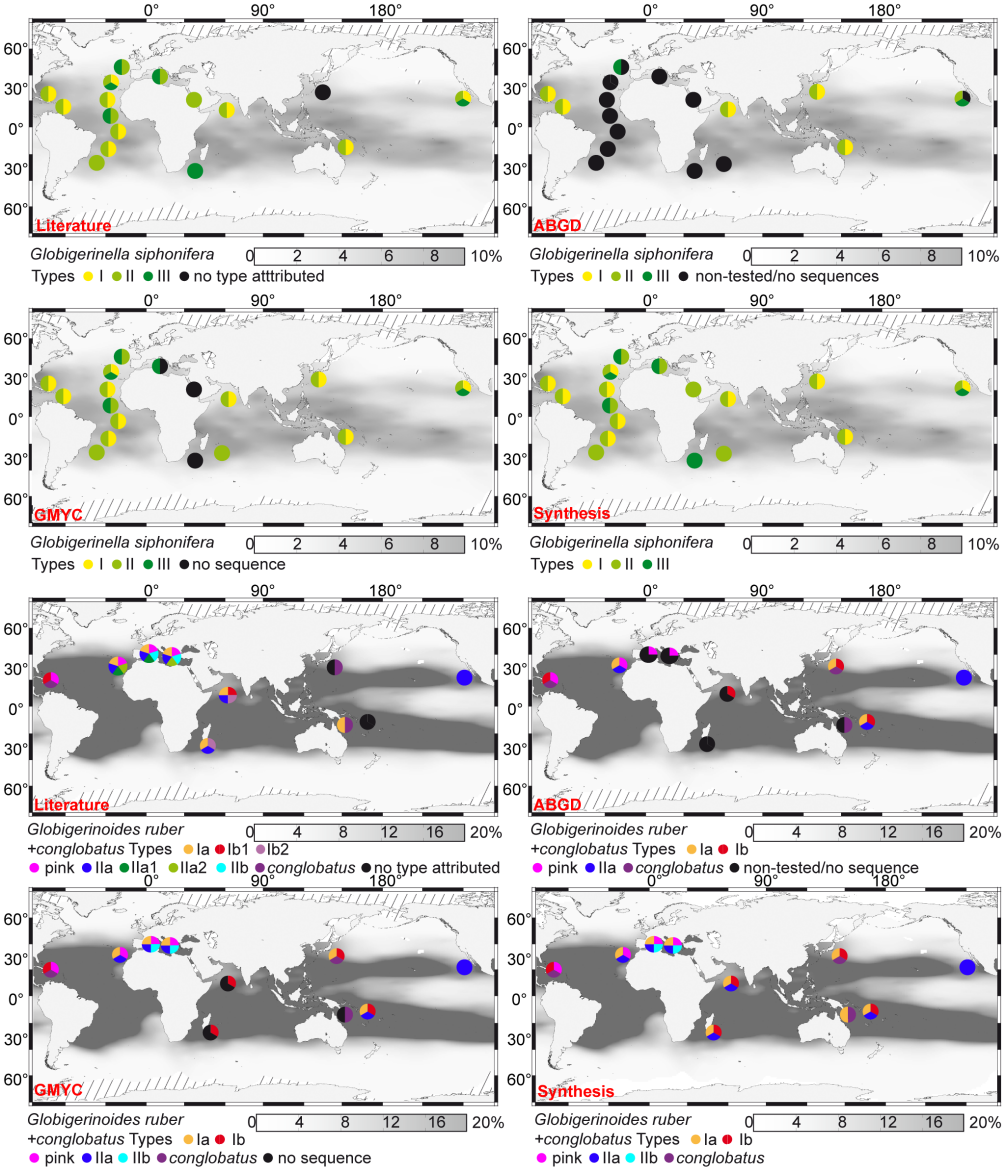
Geographic distribution of genetic types of *Globigerinella siphonifera* and *Globigerinoides ruber*. Gray shading indicates the relative abundance of each morpho-species in planktonic foraminiferal assemblages from surface sediments, interpolated from data in the MARGO database [2], [79]. The species delimitations are according to the literature and the method noted in red in the left corners of the maps. The fourth map corresponds to a synthesis. Geographic location data from [9], [13], [16], [17], [29], [31], [32], [33], [36], [39], [45], [60], [80], [81].

In seven morpho-species, there is a clear agreement between automatic and literature (pseudo)cryptic species delimitations (Table 1). For example, in *Globigerinella siphonifera*, all methods converge on the species status of the previously defined genetic Types I, II and III [31], [36], [60]. The delimitation and distributions of these types remains unchanged since the review by [9], despite our addition of new sequences and collection points (Fig. 5). Although the three genetic types exhibit wide biogeographic distributions in the world oceans, their species status is reinforced on the basis of their observed distribution pattern, which suggests ecological differences that seem to be related to nutrient availability of water masses (Fig. 5; [31]). The Type I species is apparently a surface-dweller from low nutrient water masses of the Indian, Pacific and Atlantic Oceans. The cosmopolitan Type II species has been collected in tropical to transitional water masses of the world oceans; this species may have a deeper depth habitat than the other cryptic species, since laboratory culture and plankton collections suggest that it could favor the deep chlorophyll maximum layers of the water column [31], [36], [61]. Finally, the Type III species is also a surface-dweller, but it is apparently associated with highly productive transitional water masses and upwelling systems. Species of *G. siphonifera* may be pseudo-cryptic, since [36] evidenced clear differences in shell porosity between Type I and Type II specimens collected from the Caribbean. The interpretation of this non-molecular evidence stays unchanged with the addition of a few specimens and collection points since the review by [9] (Fig. 5). In *Globigerinoides sacculifer* (1 species), *Orbulina universa* (3 species), *Hastigerina pelagica* (3 species), *Neogloboquadrina incompta* (2 species), *Neogloboquadrina dutertrei* (1 species) and *Globoconella inflata* (2 species), there is also an agreement between automatic and literature delimitations (see Text S1).

In three morpho-species, our data show that both automatic methods favor a species delimitation that does not match those available from the literature, and the resulting molecular taxonomy we propose here has repercussions on our knowledge of species biogeography. In *Globigerinoides ruber+conglobatus*, for example, our model validates the species status of only six out of the nine genetic types previously identified [9], [45]. The sympatric Types IIa, IIa1 and IIa2 [45], which appear widely distributed in tropical to transitional waters of the world ocean, are lumped into a single species here termed Type IIa (Fig. 5; Table 1). The tropical Type Ib1 and tropical to transitional Type Ib2 [9], found in sympatry in the Arabian Sea, are also lumped into a single species here termed Type Ib. The peculiar color of *G. ruber* (pink) reinforces the species status of this genetic type. Types IIa and IIb of *G. ruber*, together re-named *Globigerinoides elongatus* after [29], are distinguished from other *G. ruber* genetic types thanks to their flattened final chamber. Morphological criteria can further be argued to reinforce the species status of Type IIb, which has significantly smaller shells than other *G. ruber* species [45], and of Type IIa, which is the only large-sized species of *G. elongatus* [29]. The distinct species status of *G. conglobatus* is supported by its divergent morphological features which promoted its classification as a separate morpho-species [62]. Our revised species delimitation in *G. ruber+conglobatus* suggests that these species are nearly sympatric in tropical to transitional water masses (Fig. 5). On the other hand, different timing of reproduction evidenced by [17] between the Type Ia and *G. ruber* (pink) may suggest ecological and/or behavioral differences among species of the group. Kuroyanagi et al. [39] evidenced slight differences in stable isotopic values between shells of the Types I and II of *G. ruber*, suggesting a deeper depth-habitat (up to ∼30 m) for the latter types. Unfortunately, there is still no evidence of depth partitioning between genetic types belonging to the same clade. In *Turborotalia quinqueloba* (2 species) and *Globigerina bulloides* (7 species), our procedure also suggests that the number of (pseudo)cryptic species may have been over-estimated (see Text S1).

**Figure 6:**
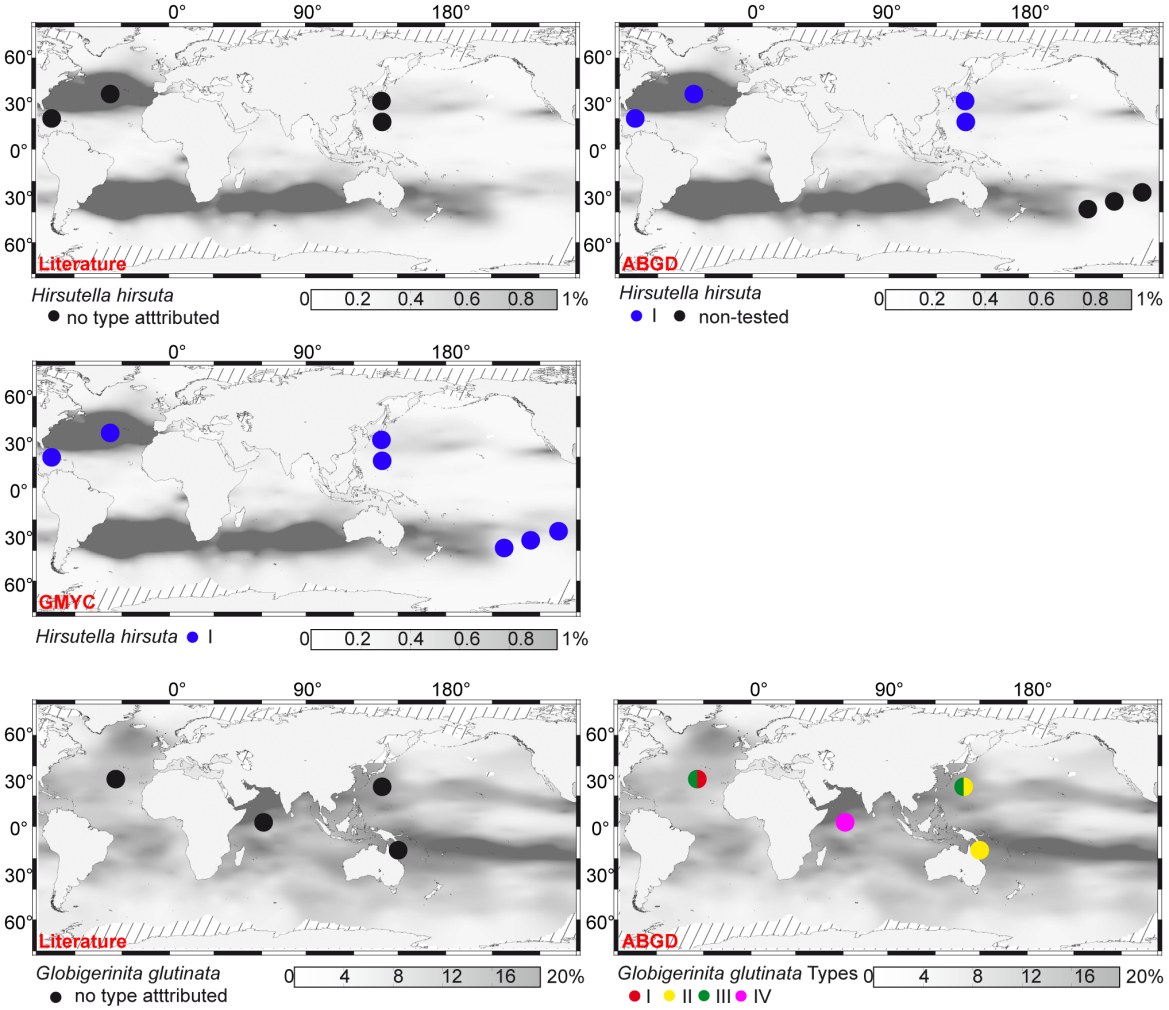
Geographic distribution of genetic types of *Hirsutella hirsuta* and *Globigerinita glutinata*. Gray shading indicates the relative abundance of each morpho-species in planktonic foraminiferal assemblages from surface sediments, interpolated from data in the MARGO database [2], [79]. The species delimitations are according to the literature and the method noted in red in the left corners of the maps. For *G. glutinata*, only delimitations according to the ABGD method are reported as genetic type delimitation by the GMYC method lead non-significant results. Geographic location data from this study and from [13], [17], [16], [32], [81].

The application of automatic methods allows us to propose, for the first time, species delimitation in several morpho-species of planktonic foraminifera (Table 1). Our data suggest that some of these morpho-species are composed of several cryptic species (4 species in *Globigerinita glutinata*, see below; 2 species in *Globigerinita uvula*, see Text S1), when others may lack cryptic diversity (*Beella digitata*, *Sphaerodinella dehiscens, Hirsutella hirsuta, Globoquadrina conglomerata, Menardella menardii, Globorotalia ungulata, Globorotalia tumida* and *Candeina nitida*). For example, ABGD splits the sequences of the microperforate *G. glutinata* into four distinct clusters (Table 1), whose patristic distances are compatible with a species status (Fig. 2B). The here defined Type I of *G. glutinata* has been collected in the subtropical north Atlantic, the Type II in the subtropical NW and SW Pacific, the Type III in the subtropical north Atlantic and NW Pacific, and the Type IV in the Arabian Sea only (Fig. 6). Considering the tropical to polar waters geographic range of this morpho-species [5], we speculate that yet un-sampled cryptic species of *G. glutinata* may inhabit colder high-latitude surface waters. On the contrary, our data show that to date, no cryptic species have been sampled in the oceans for the morpho-species *Hirsutella hirsuta* (Table 1). Considering the solid sampling effort achieved for this morpho-species in the Indian, Pacific and Atlantic Oceans (Fig. 6), we speculate that *H. hirsuta* may be a single, cosmopolitan species of planktonic foraminifera.

**Figure 7:**
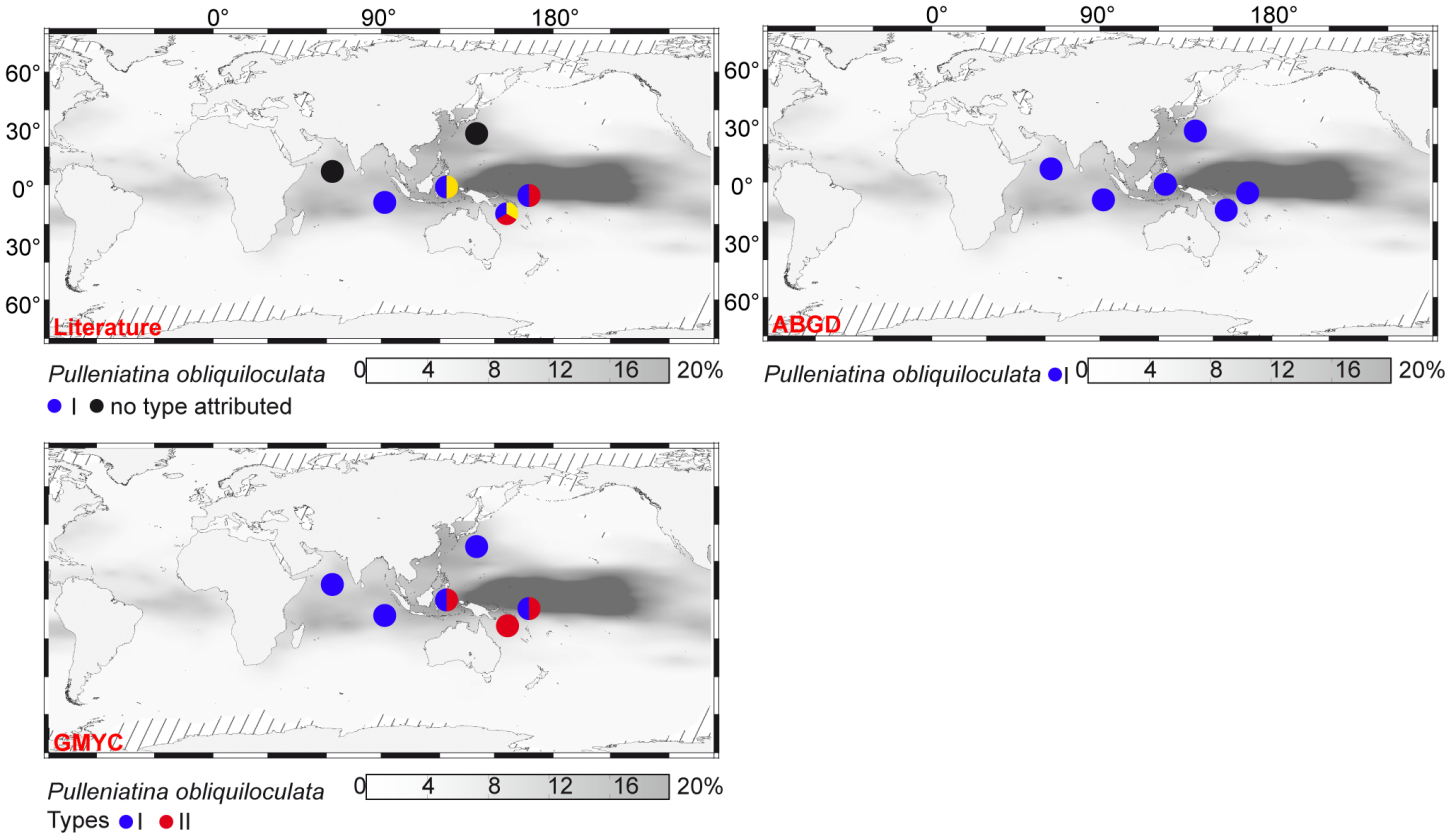
Geographic distribution of genetic types of *Pulleniatina obliquiloculata*. Gray shading indicates the relative abundance of the morpho-species in planktonic foraminiferal assemblages from surface sediments, interpolated from data in the MARGO database [2], [79]. The species delimitations are according to the literature and the method noted in red in the left corners of the maps. Geographic location data from [16], [19], [82].

Finally, many aspects of our proposed integrative taxonomy are not fully resolved. In three morpho-species (*Pulleniatina obliquiloculata* [Fig. 7], *Neogloboquadrina pachyderma, Truncorotalia truncatulinoides*, see Text S1), patristic distance distributions suggest that although they are not identical, both automatic delimitations and delimitations according to the literature are compatible with a species status. In the case of *Pulleniatina obliquiloculata*, former genetic sequencing studies have identified three genetic types [19] but the use of ABGD and GMYC suggests that those three genetic types should be lumped into one or two species (Table 1). Patristic distance approach fails to cross-validate any of these delimitations (Fig. 2). According to GMYC and to literature-based delimitations, the Type I of *P. obliquiloculata* is an independent species, occurring in tropical waters of the Indian and western Pacific Oceans (Fig. 7). While the distribution of the Types IIa and IIb are partly not overlapping [19], GMYC suggests that they should constitute a single species, here termed Type II. In the warmer waters from the tropical western Pacific, the Types I and II are found in sympatry, without any depth partitioning [19]. According to ABGD, *P. obliquiloculata* should be regarded as a single species in the Indo-Pacific.

## Conclusions

Two independent and quantitative methods, ABGD and GMYC, coupled with calculation of patristic distances within and among genetic types, were tested to quantify and compare SSU rDNA divergences in planktonic foraminifera. In *Globigerinella siphonifera* (3 species), *Globigerinoides sacculifer* (1 species), *Orbulina universa* (3 species), *Hastigerina pelagica* (3 species), *Neogloboquadrina incompta* (2 species), *Neogloboquadrina dutertrei* (1 species) and *Globoconella inflata* (2 species), we identified the same (pseudo)cryptic species as previously delimitated in the literature. In *Turborotalia quinqueloba* (2 species), *Globigerina bulloides* (7 species) and *Globigerinoides ruber+conglobatus* (6 species), our procedure suggests that only 15 out of the 26 previously described genetic types qualify a species statues. Species delimitations within *Truncorotalia truncatulinoides* (4 species), *Neogloboquadrina pachyderma* (4 species) and *Pulleniatina obliquiloculata* (2 species) remain ambiguous, although our analyses suggest that the number of (pseudo)cryptic species within these morpho-species may have been over-estimated. In the sampled and sequenced material of *Beella digitata*, *Sphaerodinella dehiscens, Hirsutella hirsuta, Globoquadrina conglomerata, Menardella menardii, Globorotalia ungulata, Globorotalia tumida* and *Candeina nitida*, we find no evidence of cryptic diversity. Finally, our data suggest for the first time that there is a cryptic diversity in *Globigerinita uvula* (2 species) and *Globigerinita glutinata* (4 species).

Whenever delimitations are ambiguous or several hypotheses are possible, calculation of patristic distances helps choosing the most plausible delimitation by maximizing inter- versus intra-type differences. Nevertheless, our analyses show that heterogeneity of the sequence dataset is detrimental for objective species delimitation. The ABGD method requires long enough and strictly overlapping sequences. Consequently, the design of the ABGD dataset excluded part of the original information, i.e., potential cryptic species. Even if the GMYC method requirements are less strict, our study shows that heterogeneity in length and/or quality of the sequences can lead to non-significant results pointing to unreliable species delimitations. Furthermore, in the case of GMYC, more sequencing effort is required to get significant species delimitations or to conclude on the taxonomic status of some of the singletons defined by this method. We note in addition that the extent of intra-genomic diversity, which may mask recent speciation events, remains unfortunately poorly investigated in planktonic foraminifera.

Our study shows that running automatic delimitation of planktonic foraminiferal cryptic species requires a high level of dataset quality. Sequences should ideally cover the six variable regions of the 1200 bp of the end part of the SSU. For each genetic type, a number of clones should be sequenced from a few individuals in order to estimate the extent of intra-genomic distances and to detect possible species over-splits whenever the automatic methods separate clones from the same individuals into different putative species. Yet, the development of automatic species delimitation methods is still at the beginning, and new and user-friendly methods are flourishing. Fast and accurate species delimitation methods have to be expected in the near future, that would, in particular, integrate non-concerted evolution as a parameter.

The species distribution of modern planktonic foraminifera is complex, with some species being cosmopolitan, and other being narrower specialists. Sampling sites remain in most cases limited, studies of seasonal distribution variations are rare, and the vertical distribution of species in the water column remains largely unknown, preventing a finer understanding of the ecology (e.g., niche partitioning) and biogeographical history of these species, especially when they are found sympatrically. From that point of view, sequencing of environmental DNA and RNA represent a great opportunity to better characterize the distribution of planktonic foraminiferal cryptic species. Such datasets are bound to multiply as Next Generation Sequencing (NGS) methods are becoming more commonplace. Nevertheless, the correct interpretation of environmental NGS datasets first needs accurate species definitions, i.e., an unambiguous reference database connecting sequences to identified genuine biological species, as promoted by this analysis.

## Material and Methods

### Ethics statement

The field collections carried out for the purpose of this paper did not involve endangered or protected species. The sampling was carried out in the open ocean and followed the regulations for the exclusive economic zones (EEZ) of the coastal countries, provided for each expedition by the respective authorities (for exact location of sampling stations, see Table S2). No specific permission was required to collect the analysed plankton.

### Material

Planktonic foraminiferal SSU rDNA data were extracted from the NCBI query portal (http://www.ncbi.nlm.nih.gov/gquery) on January 17^th^, 2013, representing a total of 1232 SSU rDNA sequences. Additional SSU rDNA data were gathered from living foraminifera we collected with plankton tows (63 to 100 µm mesh sizes) in the North Atlantic (cruises C-MarZ and FORCLIM7), Pacific (cruises REVELLE, KT-06 and GYRAFOR-A) and Indian (cruises OISO-4, OISO-19, Melville and GYRAFOR-B) Oceans (Fig. 1). During these cruises, specimens were taxonomically identified at the morpho-species level before being individually isolated into the GITC* DNA extraction buffer [37]. Information about morpho-species assignment and sampling locality is provided in Table S2.

### DNA extraction, amplification and sequencing of newly assembled data

DNA extraction of 63 collected planktonic foraminifera belonging to 12 morpho-species were performed using the GITC* extraction protocol [37]. For 34 specimens, a fragment of ∼1000 bp located at the 3′ end of the SSU rDNA was amplified using the foraminiferal specific primer S14F1 (5′-AAGGGCACCACAAGAACG C-3′) or S14p (5′-AAGGGCACCACAAGMGCG-3′) coupled with the universal primer SB (5′ GATGCCTTGTTACGACTTCTCTTTC 3′) [16]. For the other 29 specimens, a shorter fragment of ∼650 bp, within the S14F1/S14p-SB region, was amplified using the couple of specific primers S15rF (5′ CATGGCCGTTCTTAGTTC 3′)-S19F (5′ CCCGTACRAGGCATTCCTAG 3′) [21]. For the morpho-species *Globorotalia ungulata*, *Globorotalia tumida*, *Menardella menardii*, *Globoconella inflata*, *Globoquadrina conglomerata* and *Truncorotalia truncatulinoides*, the amplified PCR products were cloned using the TOPO TA cloning kit of Invitrogen following manufacturer's recommendations. To evaluate intra-individual variability, 2 to 7 clones were selected per individual for sequencing. For the morpho-species *Globigerina bulloides*, *Beella digitata*, *Orbulina universa* and *Sphaeroidinella dehiscens*, the amplified PCR products were directly sequenced. The clones and PCR products were sequenced using an ABI prism 3100 sequencer (Applied Biosystems) at the Station Biologique de Roscoff and an ABI prism 3730XL sequencer (Applied Biosystems) from Biofidal service provider (Vaulx-en-Velin, France). The new sequences obtained in this study were deposited in Genbank with accession numbers KJ633126 to KJ633260.

### Synonymy, ambiguous assignation and dataset assembly

From the 1232 SSU rDNA sequences downloaded from NCBI, 5 had no taxonomic assignation at a species rank and were not retained for further analyses. The 12 sequences labeled *Globigerinella aequilateralis* were renamed *Globigerinella siphonifera* following [5]. The 9 sequences corresponding to the right-coiled morphotype of *Neogloboquadrina pachyderma* were renamed *Neogloboquadrina incompta* following [46]. Finally, because of the polyphyletic nature of sequences from specimens identified as *Globigerinoides ruber* [45], we clustered *Globigerinoides ruber* and *Globigerinoides conglobatus* as a single taxonomic unit here termed “*G. ruber+conglobatus*”. Several sequences were found to be highly divergent from other sequences attributed to the same morpho-species and/or identical to sequences attributed to a different morpho-species. Sequence Z69600 assigned to *Globigerinoides sacculifer* in NCBI was more probably obtained from a *G. conglobatus* individual. Sequence AY453134 stored as *Globorotalia crassaformis* is identical to sequences of *Globoconella inflata.* Sequences Z83960 and JQ743485 recognized as *Globigerinella calida* are identical to sequences of one of the genetic type of *G*. *siphonifera*. Among the 6 sequences assigned to *Globorotalia scitula*, sequences FJ643321, FJ643380 and FJ643381 were found to be identical to sequences of *Hirsutella hirsuta.* Since horizontal gene transfer is rare in eukaryote taxa and has never been evidenced for rDNA [52], we consider that these seven sequences have been misassigned because of taxonomic misidentification [17] or because of possible primary or secondary contamination. These sequences were thus excluded from subsequent analyses. Following [17] and due to ambiguous identification, we also excluded from our analyses the remaining 3 sequences stored as *G. scitula*.

We thus retained 1217 sequences from NCBI. Adding the 135 newly obtained sequences, we finally constituted a dataset of a total of 1352 sequences belonging to 25 morpho-species (Table S2; Table 3). Due to differences in scientific objectives and amplification strategies, the assembled dataset contains sequences of various lengths (from 196 to 3412 bp) which do not overlap systematically. As a consequence, we defined a block of sites representing the best compromise between sequence length and number of sequences. This block of ∼700 bp, located between the universal primers SR2 ([63]; position 1277–1263 in *Saccharomyces cerevisiae*, 5′-GGTGGTGCATGGCCGG-3′) and BMB-CR ([64]; position 1624–1646 in *S. cerevisiae*, 5′-CGACGGGCGGTGTGTAC-3′), covers the variable fragments 41/f, 43/e, 45/e and 47f [65] and is documented for 714 sequences.

### Patristic distances

For each morpho-species, sequence alignments were separately and automatically generated using the Mafft software [66]. Best substitution models were determined using Modeltest (File S1; [67]), and most likely phylogenetic relationships were reconstructed using PHYML [68]. Patristic distance (path length between tips in the most likely topology) matrices were then calculated using the APE package of R software [69], [70]. Patristic distances were preferred over pairwise distances to better account for multiple substitutions at the same site. For some of the studied morpho-species, as for example *G. ruber* and *G. sacculifer*, many of the available sequences were identical while for others, as for example *Turborotalia quinqueloba* and *Neogloboquadrina pachyderma*, almost all the deposited sequences were different. This phenomenon is probably an artifact reflecting the number of sequences available, the quality of sampling and/or an arbitrary selection performed by the authors for sequences to be deposited. As a consequence, in our calculations, we retained a unique sequence per pool of identical sequences. We calculated patristic distances within each morpho-species and extracted distances within the defined genetic types and among individuals from different genetic types. We also calculated intra-genomic distances (i.e., distances observed among clones of the same individual) for each morpho-species. Since this calculation only requires individual and morpho-species identifications, sequences that lack information on their genetic type in NCBI were added to the analysis. Considering the lack of several genetic type labels in NCBI and the shortness of many sequences, it appears finally that the dataset used for calculation of patristic distance values was reduced to less than 40% (395 sequences on a total of 1352) of the available sequences (Table 3). In particular, all sequences of the Type II of *Orbulina universa* [30], and all sequences of *Globoconella inflata* with identified genetic types were excluded from our analysis. In addition, cryptic diversity has not been previously studied in the case of several morpho-species (e.g., *Globorotalia tumida* and *Globoquadrina conglomerata*). For comparing intra-genetic type, inter-genetic type and overall patristic distance distributions, we used the Kolmogorov-Smirnov and Mann-Whitney tests implemented in PAST v. 2.17c [71].

Distances between genetic types (inter-type distances) are expected to be higher than distances within genetic types (intra-type distances) whenever the genetic types correspond to genuine species. In this case, inter- and intra-type distances are separated by a distance gap (e.g., [41], [42]). Distances within individuals (intra-genomic distances) should be included in the intra-type distance range. Consequently, whenever a morpho-species displays these distance patterns, we consider here its genetic types as most probably accurately delimitated.

### Automatic genetic type delimitation methods

Genetic type delimitations used for the patristic distance approach evolved from the application of a body of various methods displaying advantages and pitfalls (e.g. those methods rely on prior species delimitation hypotheses). To avoid this, we used the Automatic Barcode Gap Discovery (ABGD; [42]) and General Mixed Yule Coalescent (GMYC; [43]) methods as independent and complementary approaches for species delimitation in modern planktonic foraminifera. ABGD and GMYC newly-defined delimitations were evaluated by running the patristic distance approach again; finally, we consider as most probably accurate the delimitations that are confirmed with the patristic distance approach.

#### Automatic Barcode Gap Discovery (ABGD)

ABGD is an automatic procedure that sorts sequences into putative species based on a barcode gap, i.e., the gap in genetic distances distribution between intraspecific and interspecific diversity [42]. This barcode gap is observed whenever the divergence among organisms belonging to the same species is smaller than divergence among organisms from different species. ABGD is more efficient when the number of species is not too large and when the inter-specific evolutionary rate differences are limited [42]. Consequently, given the particularly heterogeneous rates of molecular evolution in planktonic foraminifera [13], [72], we ran ABGD for each morpho-species alignment independently, on the same SSU rDNA region as treated for patristic distances calculations. We used the software default settings for gap detection and the K-80 pairwise distance [73]. As this method does not rely on a prior definition of taxonomic units, the analyzed dataset also includes the sequences that lacked genetic type label.

In some cases, the ABGD method identifies several MOTUs plateaus corresponding to decreasing number of genetic types delimitated within the dataset [42]. First, when necessary, we discarded the delimitation hypotheses that assigned clones from the same individual to distinct genetic types. Among the remaining hypotheses, we considered the first MOTUs plateau (i.e. maximum number of MOTUs) as corresponding to the putative species delimitation [42], [44]. Indeed, this first plateau is most likely marking the distance gap between intra-species distances (i.e., the shortest distances within the dataset) and the distances between the closest putative species [44], whereas the second plateau usually reflects the distance gap between clades of putative species.

#### General Mixed Yule Coalescent (GMYC)

The GMYC approach [43] identifies boundaries between evolutionary units on the basis of shifts in branching rates. Branching within species is the result of coalescent processes, whereas branching between species reflects the timing of speciation events [74]. This approach does not rely on both previous taxonomic delimitations and evolutionary model-based genetic distances. As a consequence, all planktonic foraminiferal sequences can be equally treated. The study of [75] demonstrates that GMYC can be successfully applied to multi-copy ribosomal genes. Preliminary experiments suggest that it requires a minimum number of biological species to get power of correctly rejecting the null hypothesis that all sequences belong to the same species. In our analysis, given the genetic distances encountered between planktonic foraminifera, we selected 5 subsets of sequences (each of them including 3 to 7 morpho-species) from the phylogeny published by [17] in order to avoid ambiguous alignments (Table 2): (*i*) the two clades within the spinose taxa, (*ii*) the two clades within the non-spinose taxa, and (*iii*) the microperforate clade. Multiple species alignments were automatically generated using the Mafft software [66] and checked manually for alignment errors. We retained a unique sequence per pool of identical sequences and the best substitution model was selected using Modeltest [67] under AIC criterion [76]. Ultrametric trees were generated with the BEAST software using a log-normal relaxed molecular clock [77] without calibration point. These trees were then used as an input for GMYC species delimitation using the Splits package [78] for R software [69].

## Supporting Information

Figure S1 **Ultrametric trees “Spinose A”, “Spinose B”, “Non-spinose A”, “Non-spinose B” and “Micoperforates” with GMYC species delimitations and sequences accession numbers.** The delimitation is significant for “Spinose A”, “Non-spinose A” and “Non-spinose B” (see Table 2). Colored branches correspond to GMYC clusters and outer circles correspond to names of morpho-species (outer arc) and plausible species (inner arc) (see Table 1). Symbols associated to specific colors indicate clones sequenced from the same individuals.
(PDF)

Table S1 **Results of Kolmogorov-Smirnov and Mann-Whitney tests for distance comparisons.**
(XLS)

Table S2 **Information on planktonic foraminiferal sequences available from NCBI (Sheet1) and information on sampling stations corresponding to new sequences (Sheet2).**
(XLSX)

File S1 **Sequence alignments, PhyML trees used for patristic distance calculation, BEAST ultrametric trees and patristic distance matrices.**
(ZIP)

Text S1 **Geographic distribution of genetic types of other morpho-species of planktonic foraminifera.**
(PDF)
