# Supplementary figures and images for: SSU rDNA Divergence in Planktonic Foraminifera: Molecular Taxonomy and Biogeographic Implications
Source: PLoS One. 2014 Aug 13;9(8):e104641. doi: 10.1371/journal.pone.0104641 (PMC4131912; doi:10.1371/journal.pone.0104641)

## Spinoze A

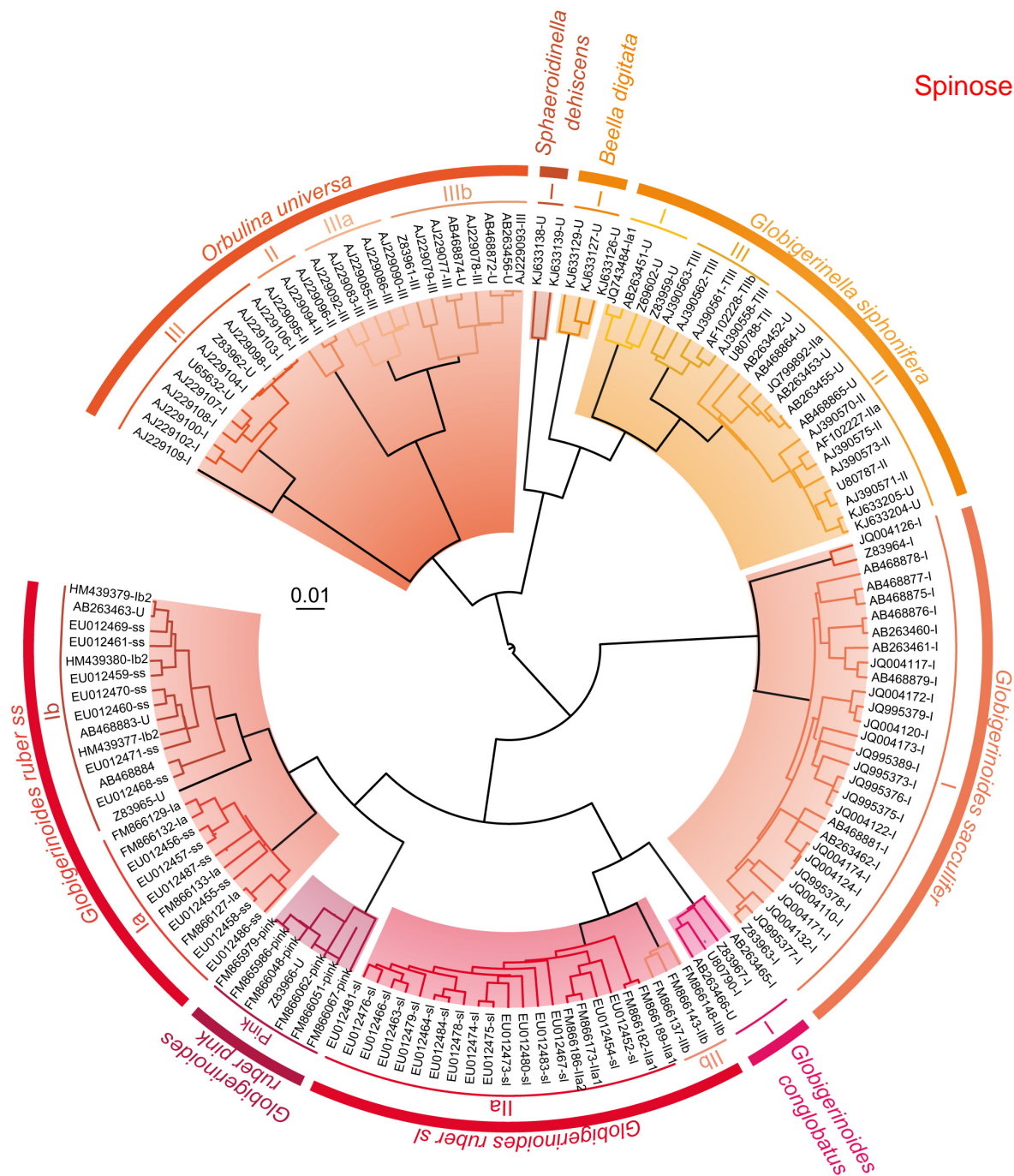

Spinose B

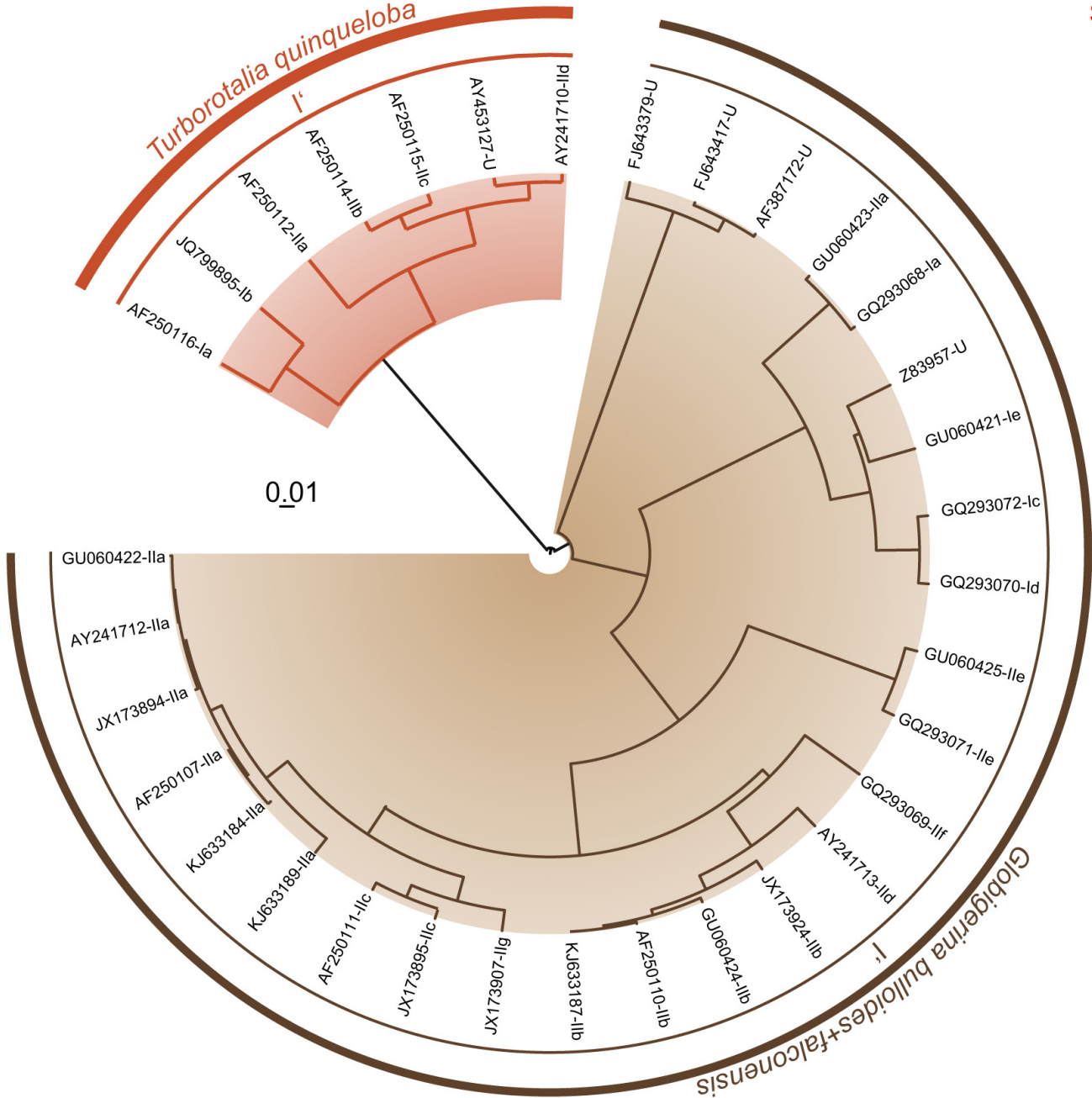

Non-spinose A

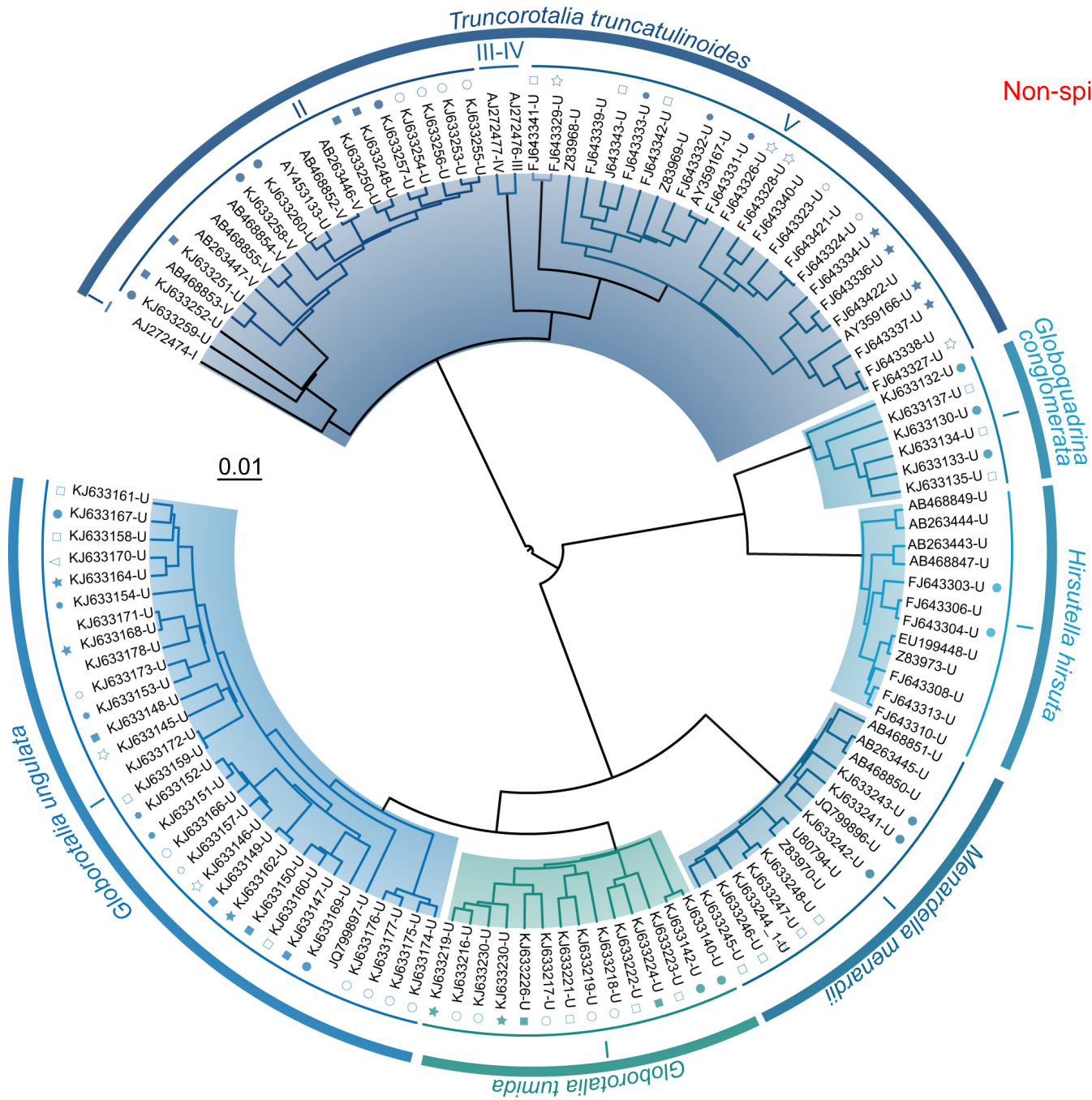

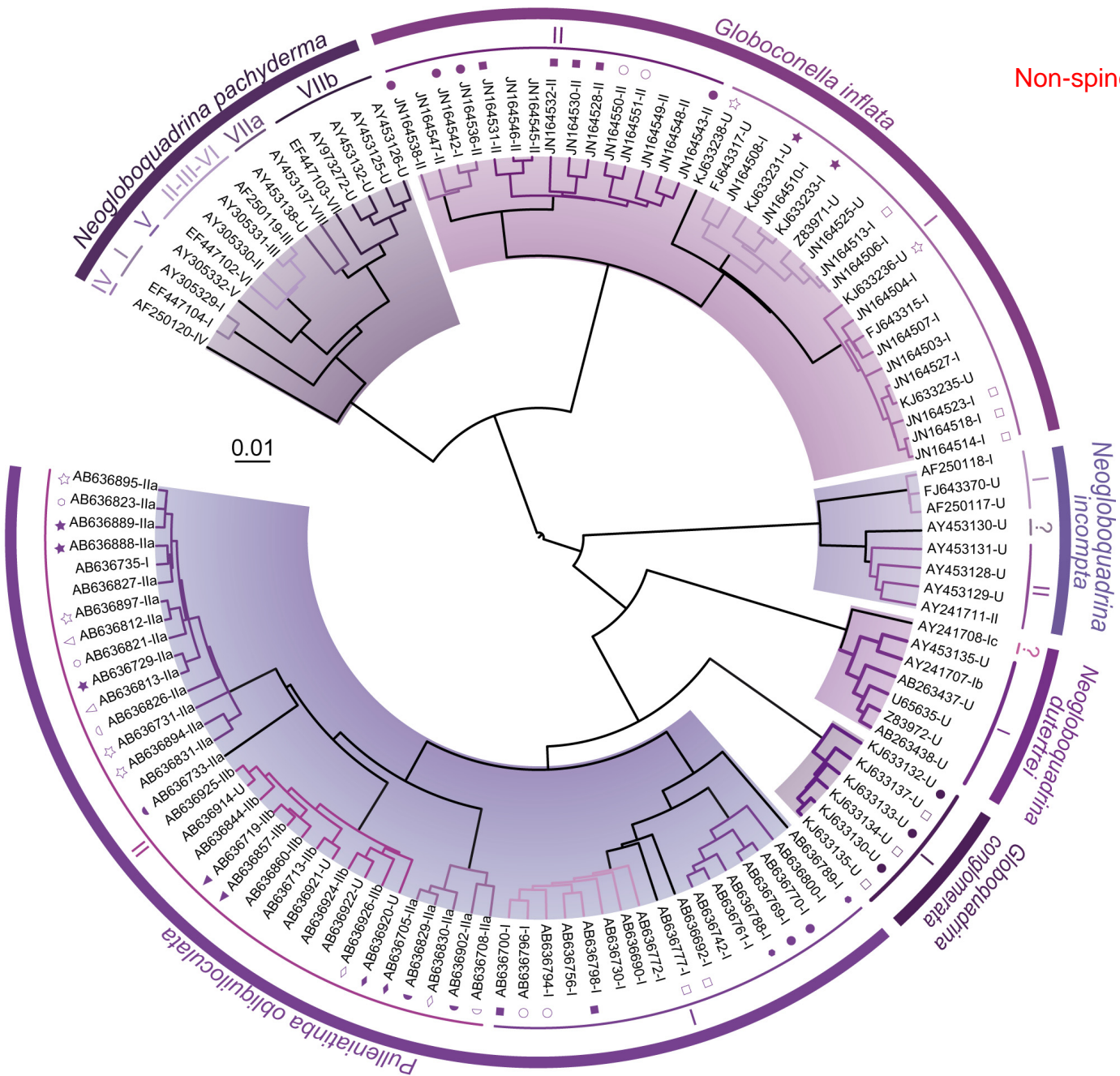

Non-spinose B

Microperforates

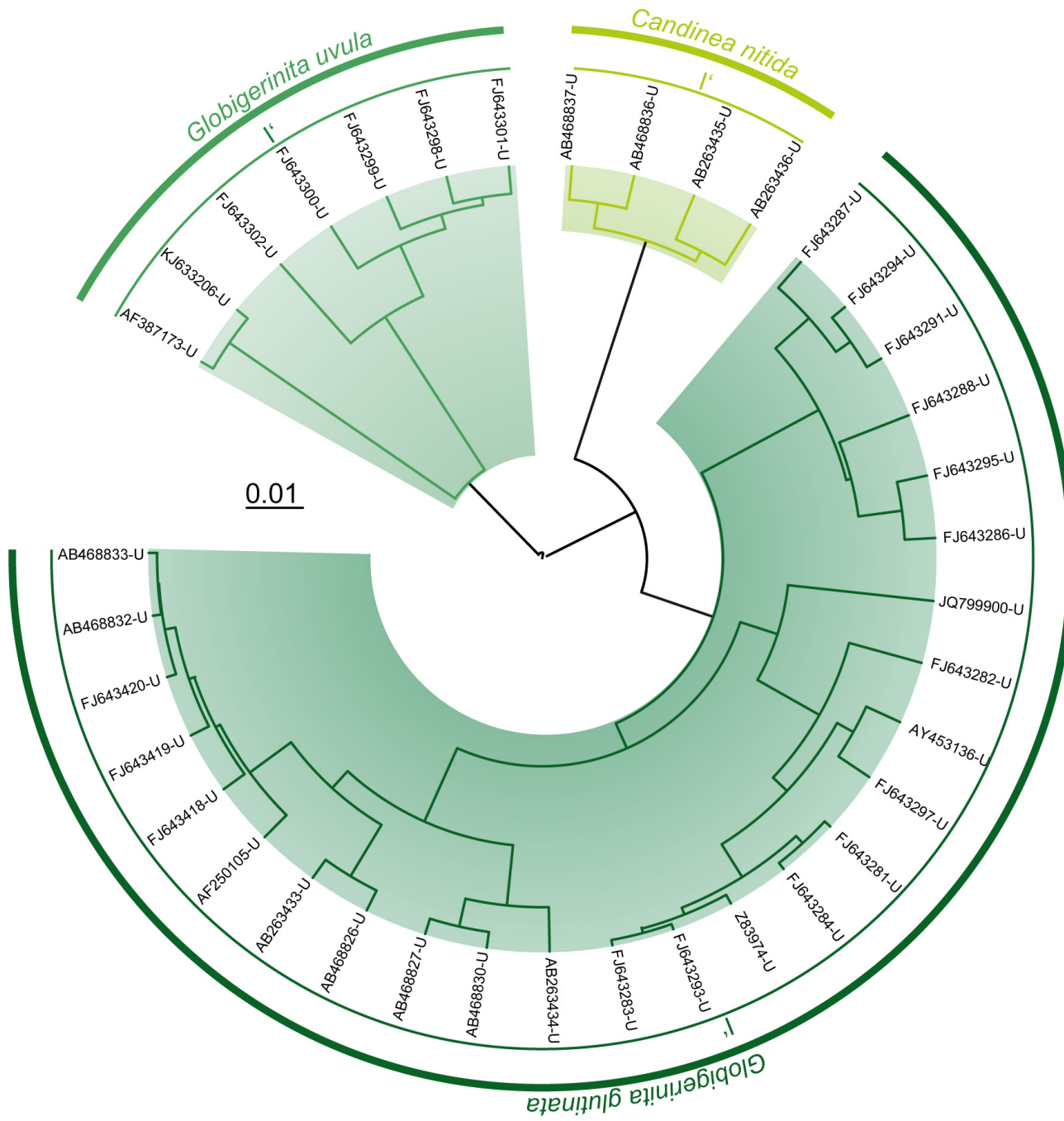

Supplement: Figure S1 — Ultrametric trees “Spinose A”, “Spinose B”, “Non-spinose A”, “Non-spinose B” and “Micoperforates” with GMYC species delimitations and sequences accession numbers. The delimitation is significant for “Spinose A”, “Non-spinose A” and “Non-spinose B” (see Table 2). Colored branches correspond to GMYC clusters and outer circles correspond to names of morpho-species (outer arc) and plausible species (inner arc) (see Table 1). Symbols associated to specific colors indicate clones sequenced from the same individuals. (PDF) [file pone.0104641.s001.pdf]
