# Supplementary material for: SSU rDNA Divergence in Planktonic Foraminifera: Molecular Taxonomy and Biogeographic Implications
Source: PLoS One. 2014 Aug 13;9(8):e104641. doi: 10.1371/journal.pone.0104641 (PMC4131912; doi:10.1371/journal.pone.0104641)
Supplement: Text S1 — Geographic distribution of genetic types of other morpho-species of planktonic foraminifera. (PDF) [file pone.0104641.s005.pdf]

**Text S1**

*SSU rDNA divergence in planktonic foraminifera: molecular taxonomy and biogeographic implications*

André A, Quillévéré F, Morard R, Ujiié Y, Escarguel G, de Vargas C, de Garidel-Thoron T, Douady CJ

Below, we provide supplementary information about the species status of modern planktonic foraminiferal genetic types, in an attempt to provide an integrative taxonomy in which we synthesize geographic, ecological and morphological differentiations that can occur among genuine (pseudo)cryptic species

<u>Spinose species</u>

For *Globigerinella siphonifera* and *Globigerinoides ruber+conglobatus*, see body-text. Patristic distances and ABGD analyses agree on the species status of the previously defined genetic Types I, IIa and IIb of *Hastigerina pelagica* (Aurahs et al., 2009a; Weiner et al., 2012). These cryptic species, collected so far in the North Atlantic, Mediterranean and NW Pacific (Fig. I; de Vargas et al., 1997; Ujiié and Lipps, 2009; Weiner et al., 2012), exhibit apparently cosmopolitan sympatric distributions in temperate to tropical water masses. Their taxonomic status is reinforced by the evidence of depth-related specializations in Types IIa and IIb (Weiner et al., 2012). The GMYC method could not be applied to *H. pelagica*, since its SSU rDNA sequences are highly divergent, leading to highly ambiguous alignments when compared with other spinose morpho-species.

Within *Turborotalia quinqueloba*, SSU rDNA studies have identified six genetic types (Darling and Wade, 2008) but the application of ABGD lumps these genetic types into two cryptic species whose patristic distances are compatible with this status (Fig. 2B). These species match the two main ecological groups evidenced by Darling and Wade (2008). The first species, here named Type I (Table 4), has been collected in Coral and Arabian Seas (Fig. II), and seems to be associated with warm waters of the (sub)tropical Indian and Pacific Oceans. The second species, here termed Type II (Table 4), exhibits a bipolar distribution in cooler (sub)polar and transitional waters of both Hemispheres (Fig. II; Darling et al., 2003). Parker (1962) reported that at polar latitudes, surface sediment samples of *T. quinqueloba* yield larger individuals than at lower latitudes. This author also noticed that specimens of *T.*

*quinqueloba* exhibit more spiny tests with latitude in the North Pacific. We speculate that there could be a relationship between these observed morphological changes that appear to follow water masses and the patchy distribution of the Types I and II of *T. quinqueloba*.

Application of the ABGD method lumps the eleven previously identified genetic types of *Globigerina bulloides* (Darling and Wade, 2008; Morard et al., 2013; Kurasawa et al., unpublished) into seven species whose patristic distances are compatible with this status (Fig. 2B). The newly defined Type IIb-d-f (Table 4) lumps sequences of individuals that apparently share similar ecology in transitional to sub-polar water masses of both hemispheres (Darling et al., 2007; Kurasawa et al., unpublished), then making Type IIb-d-f a cosmopolitan species with a bipolar distribution (Fig. II). Sequences of the Types Ic and Ie (Kurasawa et al., unpublished) from the Pacific cluster together with those of the Type Ib from the Atlantic. This suggests that the Type Ib-c-e also constitutes a cosmopolitan species. Unfortunately, since the exact sampling locations of Types Ic and Ie are not available, the ecological affinities of this species remain unknown. Data is still insufficient to evidence biogeographical or ecological differentiations between the Indo-pacific tropical Types Ia and Id. Types IIa, IIc and IIe from colder water masses also correspond to cryptic species. Type IIe is apparently restricted to the North Pacific (Darling and Wade, 2008). Darling et al. (2003) evidenced different timings of reproduction which may explain genetic isolation (Norris, 2000) of the otherwise sympatric Types IIa and IIb-d-f. Unfortunately, an integrative taxonomy for *G. bulloides* genetic types still remains limited because of the scarcity of the SSU rDNA data. For example, environmental data and sampling through different seasons are insufficient to detect potential ecological differences between the apparently sympatric Types IIc, Type IIb-d-f and Type IIa.

Our data show that only one single species of *Beela digitata* has been sampled so far. On the other hand, the distribution patterns of this rare morpho-species (Hemleben et al., 1989) cannot be discussed in this paper, since the studied sequences originate from specimens collected at two stations only, both located in the South Pacific (Fig. II).

All methods also agree on the species status of the previously defined Caribbean, Sargasso and Mediterranean genetic types of *Orbulina universa* (de Vargas et al., 1999), respectively re-designated as Types I, II and III by de Vargas et al. (2004). The ABGD method clearly delimitates the Types I and III as independent species, while sequences of the Type II were too short to be included in the ABGD dataset. The species status of the Type II is however supported on the basis of the GMYC method. In the world oceans, the three species of *O. universa* have distributions that are apparently correlated with the productivity

of the surface waters (Fig. I; de Vargas et al., 1999; Morard et al., 2009). Types I and II inhabit stratified and nutrient-depleted subtropical waters (the Type II occurring in extreme oligotrophic environments), whereas the Type III favors vertically-mixed and nutrient-rich environments of the tropical to temperate water masses. Biometric analyses by Morard et al. (2009) have shown that the Types I, II and III of *O. universa* are pseudo-cryptic species. Shells of the Type I species exhibit larger pore area and higher porosity values than those of the Types II and III, whereas the two later species can be further distinguished on the basis of shell thickness, which is significantly thinner in the case of the Type II species.

Our data confirm that the morphologically diverse plexus *Globigerinoides sacculifer*, including the *G. trilobus, G. immaturus, G. quadrilobatus* and *G. sacculifer s.s.* morphotypes, constitutes a single species (André et al., 2013). *Globigerinoides sacculifer* is so far a unique example in planktonic foraminifera where the morphological variability exceeds the rDNA genetic variability. Considering the worlwide sampling representativity of the available genetic data, *G. sacculifer* should be considered as a true cosmopolitan and morphologically diverse (sub)tropical species (Fig. I).

Finally, our data show that only one single species of *Sphaeroidinella dehiscens* has been sampled so far. Since the studied sequences originate from specimens collected at stations located in the tropical Indo-Pacific (Fig. II), the distribution pattern of this rare morpho-species cannot be discussed further (Hemleben et al., 1989).

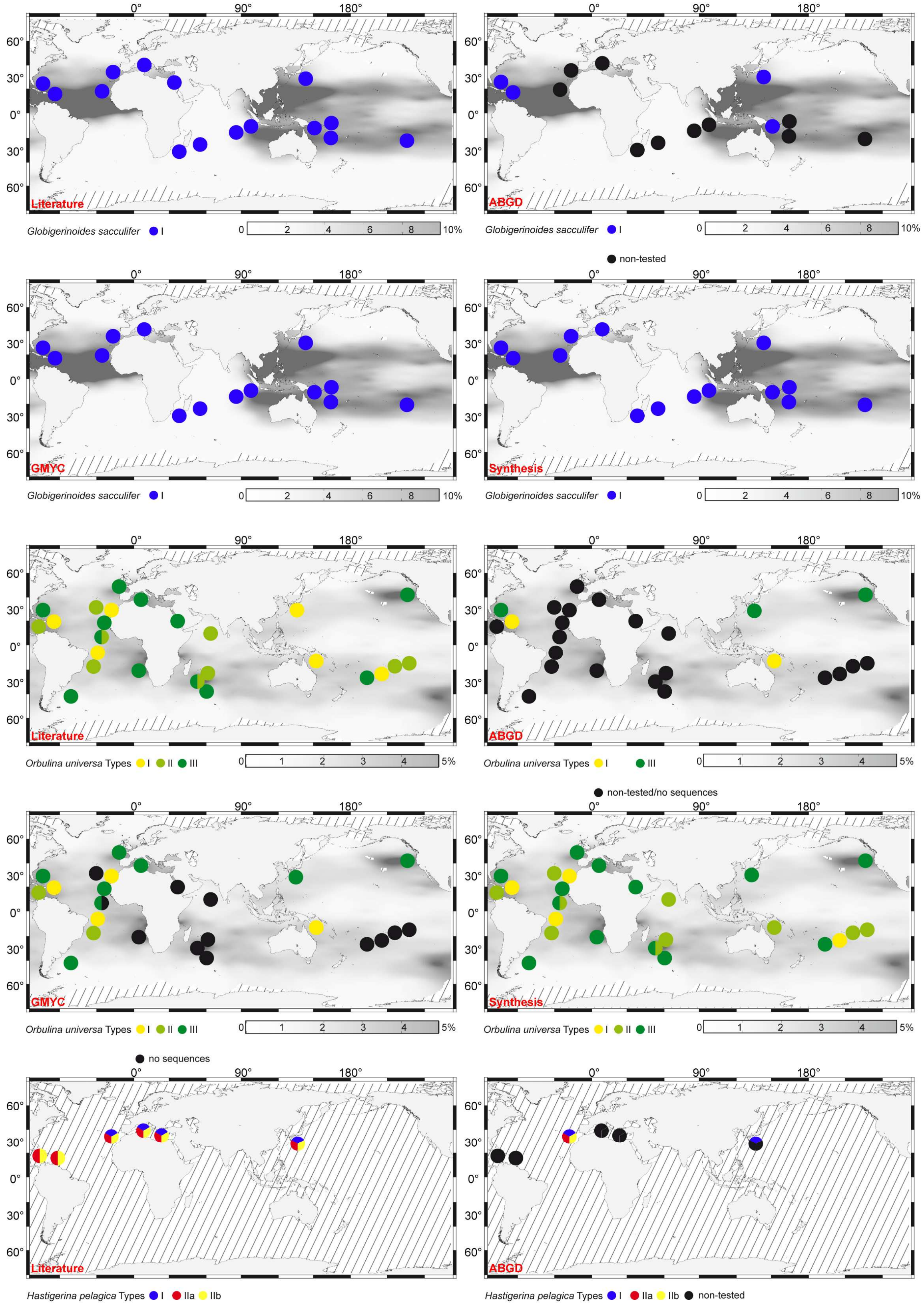


**Figure I. Geographic distribution of genetic types of *Globigerinoides sacculifer, Orbulina universa* and *Hastigerina pelagica*.** Gray shading indicates the relative abundance of each morpho-species in planktonic foraminiferal assemblages from surface sediments, interpolated from data in the MARGO database (Kucera et al., 2005; MARGO project members, 2009). The species delimitations are according to the literature and the methods noted in red in the left corners of the maps. Geographic location data from this study and Darling et al. (1997; 1999); de Vargas et al. (1997; 1999; 2004); Aurahs et al. (2009a); Morard et al. (2009); Ujiié and Lipps (2009), Weiner et al. (2012) and André et al. (2013).

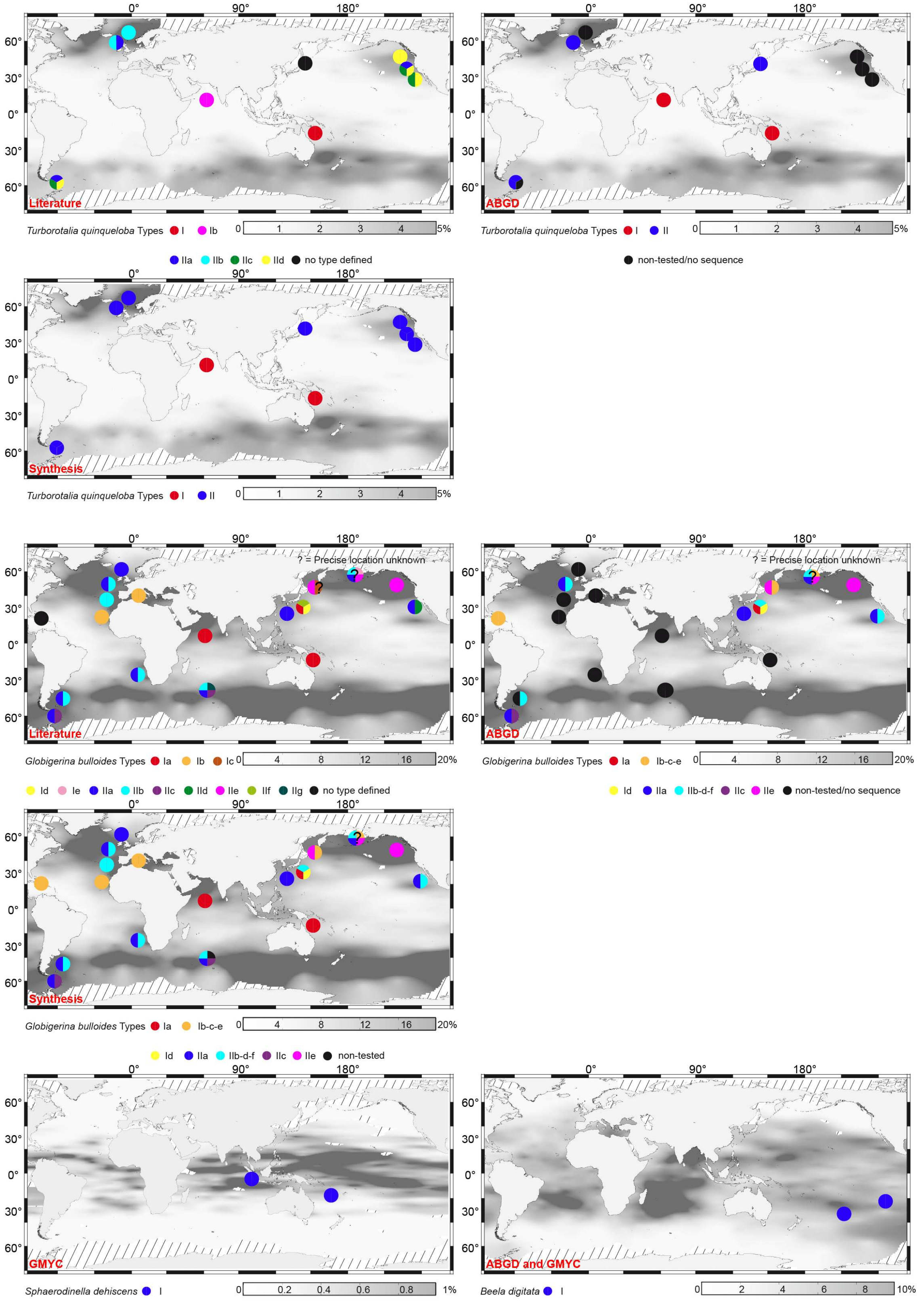


**Figure II. Geographic distribution of genetic types of *Turborotalita quinqueloba, Globigerina bulloides, Sphaerodinella dehiscens* and *Beella digtata*.** Gray shading indicates the relative abundance of each morpho-species in planktonic foraminiferal assemblages from surface sediments, interpolated from data in the MARGO database (Kucera et al., 2005; MARGO project members, 2009). The species delimitations are according to the literature and/or the methods noted in red in the left corners of the maps. Geographic location data fromthis study and de Vargas et al. (1997); Darling et al. (1999, 2000, 2003, 2007); Stewart et al. (2001); Darling and Wade (2008); Seears et al. (2012); Morard et al.(2013) ; Kimoto et al. (unpublished) and Kurasawa et al. (unpublished).

Non-spinose species

For *Hirsutella hirsuta* and *Pulleniatina obliquiloculata*, see body-text. Genetic sequencing studies have identified five genetic types within *Truncorotalia truncatulinoides* (de Vargas et al., 2001; Ujiié and Lipps, 2009), but applications of the ABGD and GMYC methods suggest that this morpho-species harbors only 3 or 4 species, respectively (Table 4). All methods agree on the species status of the right-coiled Type V (Ujiié and Lipps, 2009), which is apparently restricted to the NW Pacific where no other putative cryptic species occurs (Fig. III). ABGD is the only approach that significantly reduces intra-genetic type and inter-genetic type patristic distances overlapping (Fig. 2B). It clusters Types I and II on the one hand, and Types III and IV on the other hand (de Vargas et al., 2001), into two distinct species with contrasted environmental affinities. Type I-II is a warm-water species restricted to the (sub)tropical oceans, whereas Type III-IV appears to be restricted to the productive subtropical and the colder subpolar frontal zones of the Southern Ocean (Fig. III). Both species are pseudo-cryptic, since they can be differentiated on the basis of shell morphology: Type I-II specimens exhibit large, highly conical left or right-coiled shells, and Type III-IV specimens typically show smaller, axially-compressed and biconvex left-coiled shells (Quillévéré et al., 2013).

Our data show that to date, no cryptic species have been sampled in the oceans for the morpho-species *Hirsutella hirsuta, Menardella menardii, Globorotalia tumida, Globorotalia ungulata* and *Globoquadrina conglomerata* (Table 4). The sequences available for *M. menardii, G. tumida*, *G. ungulata* and *G. conglomerata* originate from too scarce and isolated locations of the world oceans (Fig. III), then precluding any further discussion about their cryptic diversity and biogeography.

Previous studies of the ITS genes have identified two genetic types of *Globoconella inflata* (Morard et al., 2011). ABGD and patristic distance methods agree on the species status of the Type I. However, due to the shortness of available sequences, these methods cannot be used to test whether the Type II belongs to the same species as the Type I or is indeed an independent species. Application of the GYMC method confirms the species status of the Type I, which has been found to inhabit transitional to subtropical waters of both hemispheres, and of the Type II, which is restricted to the Antarctic subpolar waters (Fig. IV). Species of *G. inflata* may be pseudo-cryptic, since Morard et al. (2011) evidenced significant differences in shell morphology in modern populations collected north and south of the Antarctic subpolar front.

Genetic sequencing studies have identified seven genetic types in *Neogloboquadrina pachyderma* (Table 1; Darling and Wade, 2008). Patristic distances are compatible with the species status of these genetic types (Fig. 2A), although the distance gap is much reduced. The distribution pattern of the genetic types defined in the literature tends to show that most of them are allopatric and related to specific water masses (Fig. V). The Type I was found in polar waters of the North Atlantic (Darling et al., 2004). The Types II and III were found in subpolar and transitional waters of the southern Ocean. The Type IV was found in Antarctic polar waters (Darling et al., 2004). The Types V and VI were only collected from the South Atlantic, in the vicinity of the Benguela upwelling (Darling and Wade, 2008). Finally, specimens of the Type VII seem to be restricted to transitional waters of the North Pacific (Darling et al., 2007). However, contrary to (Darling et al., 2004), the ABGD method leads to an alternative delimitation hypothesis which is also cross-validated by patristic distances (Fig. 2B; Table 1), clustering the genetic types from sub-antarctic and Benguela upwelling waters (i.e., Types II, III, V, VI) into a single species (Fig. V).

Regarding *Neogloboquadrina incompta*, all methods agree on the species status of the Types I and II of Darling et al. (2006). These two species have distinct distributions, the Type I inhabiting the Southern Ocean and the North Atlantic, and the Type II inhabiting the North Pacific (Fig. V). Sequence AY453130, isolated as a possible third species on the basis of GMYC analysis, is characterized by numerous substitutions that were not found in any other *N. incompta* specimens from the NW Pacific (Kimoto and Tsuchiya, unpublished), making the taxonomic status of this sequence unclear until further sampling.

Our study suggests that *Neogloboquadrina dutertrei* constitutes a unique species that is cosmopolitan in (sub)tropical waters of the world oceans (Fig. V; Table 4). The GMYC method isolated the sequence AY241708 as a putative second species (Fig. 5). Because of its short length, isolation of this sequence as a putative species may be an artifact. On the other hand, we speculate that this sequence may represent another cryptic species restricted to the North Pacific Ocean, a region that is known for harboring endemic cryptic species of planktonic foraminifera (Darling and Wade, 2008).

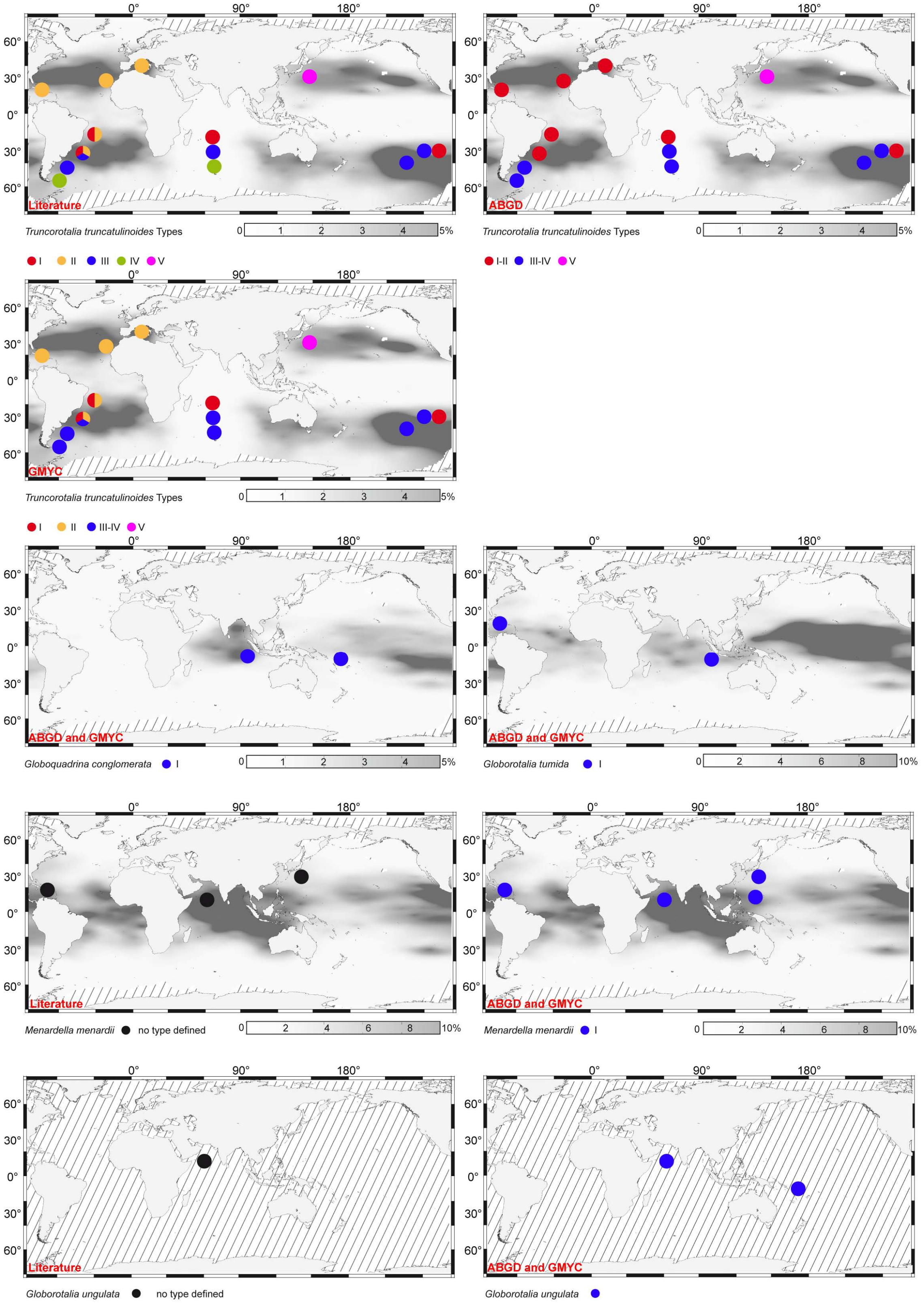


**Figure III. Geographic distribution of genetic types of *Turcorotalia truncatulinoides, Globoquadrina conglomerata, Globorotalia tumida, Globorotalia menardii* and *Globorotalia ungulata*.** Gray shading indicates the relative abundance of each morpho-species in planktonic foraminiferal assemblages from surface sediments, interpolated from data in the MARGO database (Kucera et al., 2005; MARGO project members, 2009). The species delimitations are according to the literature and/or the methods noted in red in the left corners of the maps. Geographic location data from this study and de Vargas et al. (1997; 2001); Darling et al. (1997); Ertan et al. (2004); Darling and Wade (2008); Aurahs et al. (2009a); Ujiié and Lipps (2009) and Seears et al. (2012).

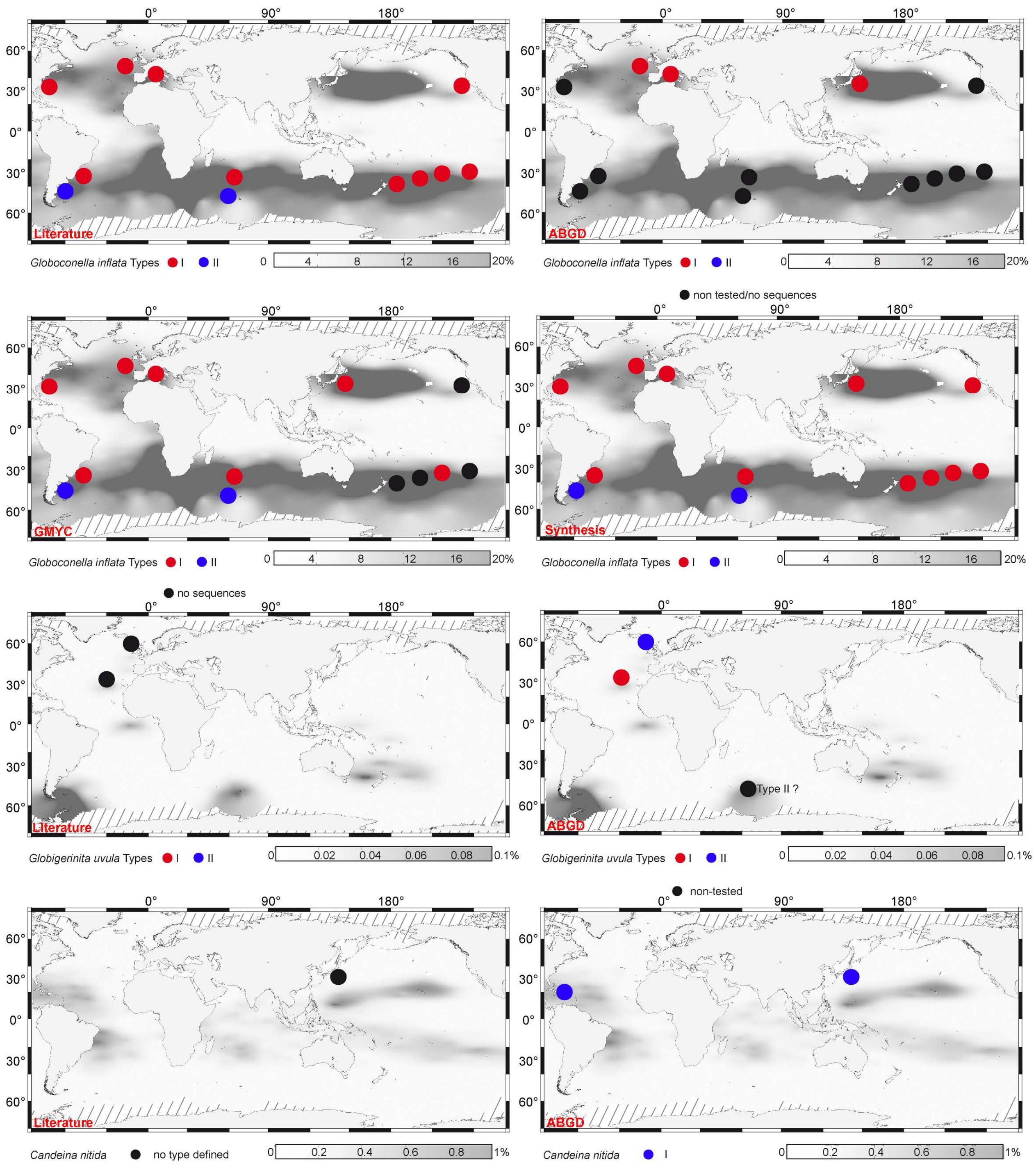


**Figure IV. Geographic distribution of genetic types of *Globoconella inflata, Globigerinita uvula* and *Candeina nitida*.** Gray shading indicates the relative abundance of each morpho-species in planktonic foraminiferal assemblages from surface sediments, interpolated from data in the MARGO database (Kucera et al., 2005; MARGO project members, 2009). The species delimitations are according to the literature and the methods noted in red in the left corners of the maps. Geographic location data from this study and de Vargas et al. (1997); Darling et al. (1999); Stewart et al. (2001); Schweizer et al. (2008); Aurahs et al. (2009a); Morard et al. (2011); Ujiié and Lipps (2009); Seears et al. (2012) and Kimoto and Tsuchiya (unpublished).

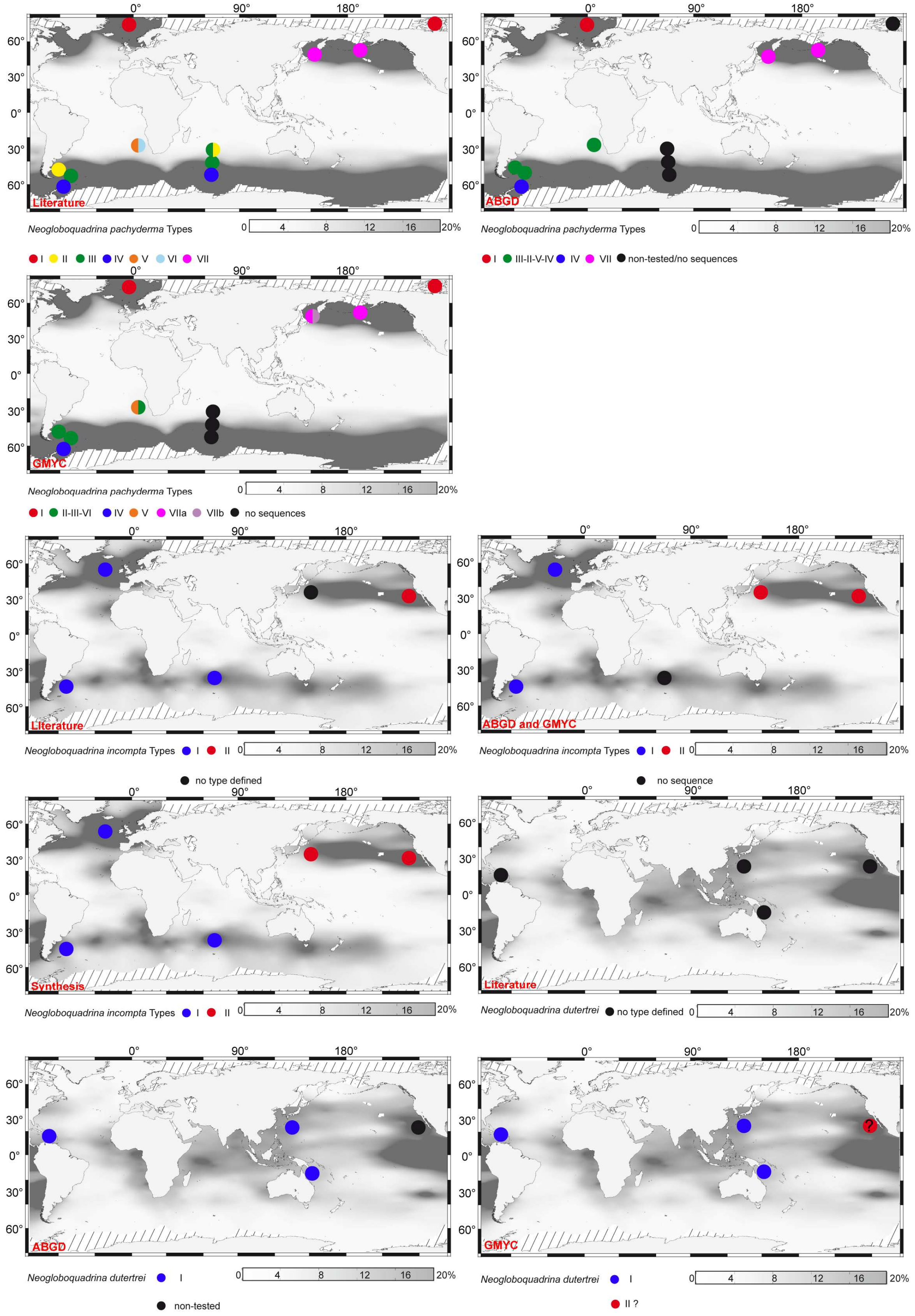


**Figure V. Geographic distribution of genetic types of *Neogloboquadrina pachyderma, Neogloboquadrina incompta* and *Neogloboquadrina dutertrei*.** Gray shading indicates the relative abundance of each morpho-species in planktonic foraminiferal assemblages from surface sediments, interpolated from data in the MARGO database (Kucera et al., 2005; MARGO project members, 2009). The species delimitations are according to the literature and/or the methods noted in red in the left corners of the maps. Geographic location data from Darling et al. (1996; 2000; 2003; 2004; 2007); de Vargas et al. (1997); Darling and Wade (2008); Schweizer et al. (2008); Aurahs et al. (2009a); Ujiié and Lipps (2009); Seears et al. (2012); André et al. (unpublished data) and Kimoto and Tsuchiya (unpublished).

Microperforate species

Up to now, no detailed genetic study has focused on the microperforate planktonic foraminifera, probably because this clade includes rare morpho-species (Hemleben et al., 1989), and because plankton net mesh and sieve sizes classically used for collecting living foraminifera are too large for these typically small-sized taxa. Here, our models for species delimitation are based solely on distance methods (patristic and ABGD), since GYMC delimitations were not significantly better than the null hypothesis of a pure coalescent model (Table 5).

For *Globigerinita glutinata*, see body-text. Application of the ABGD method on the dataset of *Globigerinita uvula* lumps sequences into two distinct clusters (Table 4) whose patristic distances are compatible with the species status (Fig. 2B). Such a species status of the Types I and II of *G. uvula* is apparently reinforced on the basis of observational geographical data, which suggest that the first species inhabits subtropical environments, when the second species may be restricted to subpolar environments (Fig. IV).

For the sequences of *Candeina nitida,* estimation of patristic distance values (Fig. 2A) suggests that only one cosmopolitan species has been sampled so far in the Caribbean Sea and NW Pacific (Fig. IV).

**References**

André, A., Weiner, A., Quillévéré, F., Aurahs, R., Morard, R., Douady, C.J., de Garidel-Thoron, T., Escarguel, G., de Vargas, C., Kucera, M., 2013. The cryptic and the apparent reversed: lack of genetic differentiation within the morphologically diverse plexus of the planktonic foraminifer *Globigerinoides sacculifer*. Paleobiology, 39: 21-39.

Aurahs, R. Göker, M., Grimm, G., Hemleben, V., Hemleben, C., Schiebel, R. and Kucera, M., 2009a. Using the Multiple Analysis Approach to Reconstruct Phylogenetic Relationships among Planktonic Foraminifera from Highly Divergent and Length-polymorphic SSU rDNA Sequences. Bioinformatics and Biology Insights, 2009: 155.

Aurahs, R., Grimm, G.W., G., Hemleben, V., Hemleben, C. and Kucera, M., 2009b. Geographical distribution of cryptic genetic types in the planktonic foraminifer *Globigerinoides ruber*. Molecular Ecology, 18(8): 1692-1706.

Aurahs, R., Treis, Y., Darling, K. and Kucera, M., 2011. A revised taxonomic and phylogenetic concept for the planktonic foraminifer species *Globigerinoides ruber* based on molecular and morphometric evidence. Marine Micropaleontology, 79(1-2): 1-14.

Bijma, J., Hemleben, C., Huber, B.T., Erlenkeuser, H. and Kroon, D., 1998. Experimental determination of the ontogenetic stable isotope variability in two morphotypes of *Globigerinella siphonifera* (d'Orbigny). Marine Micropaleontology, 35(34): 141-160.

Brady, H.B., 1879. Notes on the reticularean Rhizopoda of the Challenger Expedition, Part II: Additions to the knowledge of the porcellanous and hyaline types. The Quaterly Journal of Microscopical Science, 19: 61-299.

Darling, K.F., Kroon, D., Wade, C.M. and Leigh Brown, A.J., 1996. Molecular phylogeny of the planktic foraminifera. Journal of Foraminiferal Research, 26(4): 324-330.

Darling, K.F., Kucera, M., Kroon, D. and Wade, C.M., 2006. A resolution for the coiling direction paradox in *Neogloboquadrina pachyderma*. Paleoceanography, 21(2): PA2011.

Darling, K.F., Kucera, M., Pudsey, C.J. and Wade, C.M., 2004. Molecular evidence links cryptic diversification in polar planktonic protists to Quaternary climate dynamics. PNAS, 101(20): 7657-7662.

Darling, K.F., Kucera, M. and Wade, C.M., 2007. Global molecular phylogeography reveals persistent Arctic circumpolar isolation in a marine planktonic protist. PNAS, 104(12): 5002-5007.

Darling, K.F., Kucera, M., Wade, C.M., Langen, P.V. and Pak, D., 2003. Seasonal distribution of genetic types of planktonic foraminifer morpho-species in the Santa Barbara Channel and its paleoceanographic implications. Paleoceanography, 18(2): 1032-1042.

Darling, K.F. and Wade, C.M., 2008. The genetic diversity of planktic foraminifera and the global distribution of ribosomal RNA genotypes. Marine Micropaleontology, 67(3-4): 216-238.

Darling, K. F., C. M. Wade, D. Kroon, and A. J. Leigh Brown. 1997. Planktic foraminiferal molecular evolution and their polyphyletic origins from benthic taxa. Marine Micropaleontology 30:251–266.

Darling, K.F., Wade, C.M., Stewart, I.A., Kroon, D., Dingle R. and Brown, A.J., 2000. Molecular evidence for genetic mixing of Arctic and Antarctic subpolar populations of planktonic foraminifers. Nature, 405: 43-47.

Darling, K.F., Wade, C.M., Kroon, D., Brown, A.J.L. and Bijma, J., 1999. The diversity and distribution of modern planktic foraminiferal small subunit ribosomal RNA genotypes and their potential as tracers of present and past circulation. Paleoceanography, 14(1): 3-12.

Darling, K. F., C. M. Wade, D. Kroon, and A. J. Leigh Brown. 1997. Planktic foraminiferal molecular evolution and their polyphyletic origins from benthic taxa. Marine Micropaleontology 30:251–266.

Dayrat, B., 2005. Towards integrative taxonomy. Biological Journal of the Linnean Society, 85(3): 407-415.

De Queiroz, K., 2007. Species Concepts and Species Delimitation. Systematic Biology, 56(6): 879-886.

de Vargas, C., Zaninetti, L., Hilbrecht, H. and Pawlowski, J., 1997. Phylogeny and Rates of Molecular Evolution of Planktonic Foraminifera: SSU rDNA Sequences Compared to the Fossil Record. Journal of molecular evolution, 45: 285-294.

de Vargas, C., Norris, R.D., Zaninetti, L., Gibb, S.W. and Pawlowski, J., 1999. Molecular evidence of cryptic speciation in planktonic foraminifers and their relation to oceanic provinces. PNAS, 96: 2864-2868.

de Vargas, C., Renaud, S., Hilbrecht, H. and Pawlowski, J., 2001. Pleistocene adaptive radiation in *Globorotalia truncatulinoides*: genetic, morphologic, and environmental evidence. Paleobiology, 27(1): 104-125.

de Vargas, C., Bonzon, M., Rees, N.W., Pawlowski, J. and Zaninetti, L., 2002. A molecular approach to biodiversity and biogeography in the planktonic foraminifer *Globigerinella siphonif*era (d'Orbigny). Marine Micropaleontology, 45: 101-116.

De Vargas, C., Saez, A.G., Medlin, L.K. and Thierstein, H.R., 2004. Super-species in the calcareous plankton. Thierstein, H.R. and Young, J.R. (eds): Coccolithophores- from Molecular Processes to Global Impact. Springer, Berlin, p. 271-298.

Ertan, K., Hemleben, V. and Hemleben, C., 2004. Molecular evolution of some selected benthic foraminifera as inferred from sequences of the small subunit ribosomal DNA. Marine Micropaleontology, 53: 367-388.

Hemleben, C., Spindler, M. and Anderson, O.R., 1989. Modern Planktonic Foraminifera. Springer, New York, 363 pp.

Huber, B.T., Bijma, J. and Darling, K.F., 1997. Cryptic speciation in the living planktonic foraminifer *Globigerinella siphonifera* (d'Orbigny). Paleobiology, 23(1): 33-62.

Kucera, M., Rosell-Melé, A., Schneider, R., Waelbroeck, C. and Weinelt, M., 2005. Multiproxy approach for the reconstruction of the glacial ocean surface (MARGO). Quaternary Science Reviews, 24: 813-819.

Kurasawa, A., Tsuchiya, M., Toyofuku, T., Kitazato, H. and Nishi, H., unpublished. Genetic variability of *Globigerina bulloides* in the Eastern North Pacific subtropical region and the bipolarity of planktic foraminiferal genotypes.

Kuroyanagi, A., Tsuchiya, M., Kawahata, H. and Kitazato, H., 2008. The occurrence of two genotypes of the planktonic foraminifer *Globigerinoides ruber* (white) and paleo-environmental implications. Marine Micropaleontology, 68(3-4): 236-243.

Kimoto, K. and Tsuchiya, M., unpulished. Coiling direction changes in different generations of Neogloboquadrina pachyderma: importance on paleoceanographic reconstructions from the evidence of laboratory cultivation and molecular phylogenetic analysis.

MARGO project members, 2009. Constraints on the magnitude and patterns of ocean cooling at the last glacial maximum. Nature Geoscience, 2: 127-132.

Morard, R., Quillévéré, F., Escarguel, G., Ujiie, Y., de Garidel-Thoron, T. Norris, R. D. and de Vargas, C., 2009. Morphological recognition of cryptic species in the planktonic foraminifer *Orbulina universa*. Marine Micropaleontology, 71(3-4): 148-165.

Morard, R., Quillévéré, F., Douady, C. J., de Vargas, C., de Garidel-Thoron, T. and Escarguel, G., 2011.Worldwide Genotyping in the Planktonic Foraminifer *Globoconella inflata*: Implications for Life History and Paleoceanography. PLoS ONE, 6(10): e26665.

Morard, R., Quillévéré, F., Escarguel, G., de Garidel-Thoron, T., de Vargas, C., and Kucera, M., 2013. Ecological modeling of the temperature dependence of cryptic species of planktonic foraminifera in the Southern Hemisphere. Palaeogeography, Palaeoclimatology, Palaeoecology, 391, 13-33.

Norris, R.D., 2000. Pelagic species diversity, biogeography and evolution. Paleobiology, 26: 236-258.

Parker, F.L., 1962. Planktonic foraminiferal species in Pacific sediments. Micropaleontology, 8(2): 219-254.

Schweizer, M., Pawlowski, J., Kouwenhoven, T., Guiard, J. and Van Der Zwaan, B., 2008. Molecular phylogeny of Rotaliida (Foraminifera) based on complete small subunit RDNA sequences. Marine Micropaleontology, 66: 233-246.

Seears, H., Darling, K. and Wade, C., 2012. Ecological partitioning and diversity in tropical planktonic foraminifera. BMC Evolutionary Biology, 12 : 54.

Stewart, I.A., Darling, K.F., Kroon, D., Wade, C.M. and Troelstra, S.R., 2001. Genotypic variability in subarctic Atlantic planktic foraminifera. Marine Micropaleontology, 43(1-2): 143-153.

Ujiie, Y., Asami, T., de Garidel-Thoron, T., Liu, H., Ishitani, Y. and de Vargas, C., 2012. Longitudinal differentiation among pelagic populations in a planktic foraminifer. Ecology and Evolution, 2(7): 1725-1737.

Ujiie, Y. and Lipps, J.H., 2009. Cryptic diversity in planktonic foraminifera in the northwest Pacific Ocean. Journal of Foraminiferal Research, 39(3): 145-154.
